# Outsourcing National Health Service Surgery to the Private Sector: Waiting Time Inequality and the Making of a Two-Tier System for Hip and Knee Replacement in England

**DOI:** 10.1177/27551938251336949

**Published:** 2025-04-27

**Authors:** Graham Kirkwood, Allyson M. Pollock

**Affiliations:** 1 Population Health Sciences Institute, Faculty of Medical Sciences, 5994 Newcastle University, Newcastle upon Tyne, UK

**Keywords:** health inequities, national health service, surgery, privatization, wait time

## Abstract

This study analyzes National Health Service (NHS)-funded elective primary hip and knee replacement admissions and waiting times in England by provider (the NHS and private), socioeconomic deprivation and comorbidity, both prior to the introduction of Independent Sector Treatment Centers from 1997 to 2003 and following the rapid expansion in NHS contracts with the private sector from 2008 to 2019. Between 1997 and 2019, NHS-funded admission rates more than doubled. Between 2003 and 2008, when the proportion of patients treated in the private sector was negligible, admissions to the NHS increased and waiting times more than halved. After 2008, following the expansion in use of private providers by the NHS, NHS admission rates fell and waiting times rose for all patients. Waiting times for private providers were half those for the NHS, and the poorest 20 percent waited longer than the richest 20 percent. Between 2003 and 2019, inequalities in waiting time rose for the poorest 20 percent. The introduction of private providers into the NHS is associated with a contraction in in-house NHS provision, increasing waiting times for all patients and a two-tier system operating in favor of the rich.

At the end of November 2024 there were approximately 6.3 million patients waiting for 7.5 million treatments in the National Health Service (NHS) in England.^1^ NHS England's response to waiting lists and backlog in elective care is to “transfer…high volume and low complexity conditions, as well as some cancer pathways and diagnostics, to the independent sector”.^2^

The policy of using the private sector for NHS patients was set out in the NHS Plan 2000^3^ as a partnership between the NHS and the private sector to develop a new generation of diagnostic and treatment centers. The first tranche of independent sector treatment center (ISTC) contracts awarded in 2003 were intended to provide up to 171,000 first finished consultant episodes annually for five years at a cost of approximately £1.6 billion. The second tranche, launched in March 2005, were to provide a further 150,000 procedures a year at an overall cost of £3 billion over 5 years.^4^ The private sector was guaranteed payment for each patient referred (whether treated or not) at the agreed minimum referral value, known as “take or pay”.^3^

In 2006, the program was extended and NHS-funded patients were given a menu of services from nationally approved private providers.^5^ In April 2008 any hospital provider that met NHS standards and costs was allowed to compete for patients (see Table 1).^6–8^

**Table 1.** Timeline and Milestones.

Period 1: April 1, 1997-December 31, 2002	Election of the New Labor government on May 1, 1997. July 1999—ACAD, Europe's first dedicated elective treatment center, opened at Central Middlesex Hospital.^9^ The NHS Plan: a plan for investment, a plan for reform was published on July 1, 2000. April 2002—NHS treatment center program announced in Delivering the NHS Plan: next steps on investment, next steps on reform. December 2002—ISTC program announced in Growing Capacity: independent sector diagnosis and treatment centers.
Period 2: January 1, 2003-December 31, 2005	September 2003—first ISTC contracts were signed. Opening of the first eight ISTCs, including the pilot ISTC Redwood Diagnosis and Treatment Centre in January 2003, and the first regular ISTC, the Birkdale Clinic ISTC in October 2003. Recording of private providers by NHS Digital began in January 2003. The London Patient Choice Project extended to orthopedics, ear/nose/throat, and general surgery in April 2003 ^10^. Patients waiting more than six months for elective surgery in England were offered the choice of an alternative provider in August 2004 ^5^. March 2005—second phase of ISTCs announced.
Period 3: January 1, 2006-March 31, 2008	Patient choice launched nationally on January 1, 2006, with “extended choice network” of at least four providers to choose from ^5,7^. Another six ISTCs opened. Prior to 2006, a patient's choice of hospital was limited to those with which their local health authority had a contract ^11^.
Period 4: April 1, 2008^a^-March 31, 2019^b^	Free choice for elective treatment of any hospital provider that meets NHS standards and costs launched April 2008, further expanding the use of private providers ^5^. By the end of 2009, there were 39 surgical ISTCs in operation.

^a^ Orthopedic services went full “free choice” on July 1, 2007,^6^ however it wasn’t until April 2008 when the number of provider sites and the number of admissions to private providers significantly increased. The number of provider sites for hip and knee replacements both went from 24 in the last quarter of 2007-2008 to 61 in the first quarter of 2008-2009 (see Supplemental Table ST14 and Figure SF1). The share of all hip and knee replacements provided by the private sector went from 5.4 percent and 5.7 percent, respectively, in the last quarter of 2007-2008 to 9.9 percent and 9.6 percent in the first quarter of 2008-2009 (see Supplemental Table ST15 and Figure SF2).

^b^ The 2012 Health and Social Care Act did not extend the policy of patient choice of any qualified provider.^12^

## Waiting Times

Maximum waiting times for elective care were first introduced in 1991 in the Patients’ Charter.^13,14^ Following the election of the New Labor government in 1997, a “war on waiting” was declared.^15^ By early 2000 the target of reducing waiting lists by 100,000 patients had been achieved.

The NHS Plan 2000 aimed to abolish waiting lists and reduce the maximum wait from 18 to 6 months for outpatient appointment to inpatient treatment, with sanctions linked to targets.^16^ Chief executives of failing NHS trusts could be named and shamed and lose their jobs if targets were not met. Successful trusts were rewarded with retention of financial surpluses and less central control.^17,18^ In December 2008 a new target of 18 weeks maximum wait between referral and inpatient admission was introduced.^19^ Financial sanctions for breaching elective waiting time targets were removed in 2016.^13^

## Inequalities

Patients living in the most deprived quintiles of the population of England are more than twice as likely to need a hip replacement than those in the middle quintile, but only four fifths as likely to receive one.^20^ Reducing inequalities is an important objective for the NHS in England. Statutory duties (legal duties arising from an act of parliament) aimed at reducing inequalities were imposed on NHS bodies in 2012 and 2022, and although socioeconomic deprivation stands outside the Equality Act 2010's protected characteristics, the framework provided by the health inequalities duties^21^ include ensuring “equity of access, experience and outcomes for the most deprived 20% of the population.”

Since 2003, the proportion of NHS elective hip and knee replacements undertaken in ISTCs and private hospitals has grown.^11^ Research also shows those living in the least deprived areas of England have benefitted most from NHS-funded private provision of hip surgery.^22^ In Scotland between 2007 and 2010, outsourcing was associated with a significant fall in in-house NHS provision and increasing inequality in treatment rates for patients who were older and poorer who received elective hip replacement.^23^ Despite these findings, current UK Labor government policy is to continue privatizing NHS care in England.^24^

The aim of this study is to analyze the effect of using the private sector for NHS primary hip and knee replacements on admissions and waiting times for the most deprived 20 percent of the population in England between 1997 and 2019.

## Methods

### Data

Extracts of all NHS England-funded elective primary hip and knee replacement admissions between April 1, 1997, and March 31, 2019, purchased as Hospital Episode Statistics data from NHS Digital at a cost of £11,964 plus £2,140 for a time extension. Details of variables including provider (the NHS or private), inclusion criteria, and derivation of deprivation and comorbidity measures are provided in the Supplemental Material. Deprivation quintiles were IMD1 = most deprived and IMD5 = least deprived.

### Admissions

Numbers of admissions and admission rates were calculated by financial year and for each of the four periods (see Table 1) by provider (all providers, the NHS and private). The odds of admission for patients in IMD1 and IMD5 and with and without comorbidity were calculated by financial year and period, by provider.

### Waiting Times

#### Trends

Waiting time was calculated as days between decision to admit and admission.^25,26^ Mean waiting times were calculated by financial year and period by provider.

#### Inequality

Data were analyzed using simple linear regression with an interrupted time series design with outcome being the difference in mean waiting time per financial year quarter between patients in IMD1 and IMD5; split between pre-privatization, period 1 (April 1, 1997, to December 31, 2002), and post-privatization, periods 2, 3, and 4 combined (January 1, 2003, to March 31, 2019) (see the Supplemental Material).

#### The Impact of Private Providers on Waiting Time and Waiting Time Inequality

We examined the effect on waiting time of provider (the NHS or private), number of admissions per day and share of admissions going to private providers during a patient's wait. The latter two variables are “external variables” which exist independently of any individual (see the Supplemental Material). The model was adjusted for deprivation, comorbidity, period, and gender with an interaction between provider and deprivation.

Data were analyzed using parametric survival analysis methods as time to event data, the event of interest being admission to hospital. Loglogistic models with shared frailty on purchase code (PURCODE: organization commissioning patient's health care) were chosen (see the Supplemental Material). All variables were included in all models. Results were interpreted as time ratios^27^ used to calculate the relative percentage change in waiting times between categories and per unit for continuous variables.

There was no adjustment for age or sex “on the basis of the value judgement that (at least in most cases) age and sex are not a legitimate justification for making people wait longer for needed treatment”.^26^

## Results

### Hip Replacements

#### Admissions

There were 1,167,002 NHS-funded elective primary hip replacement admissions with waiting time less than three years between April 1, 1997, and March 31, 2019 (see Supplemental Table ST1). There were no admissions to private providers in period 1. The number and proportion of admissions to private providers in periods 2, 3, and 4 were 2,049 (1.46%), 4,648 (3.88%), and 146,812 (20.84%), respectively (see Supplemental Table ST2).

**Figure 1.**
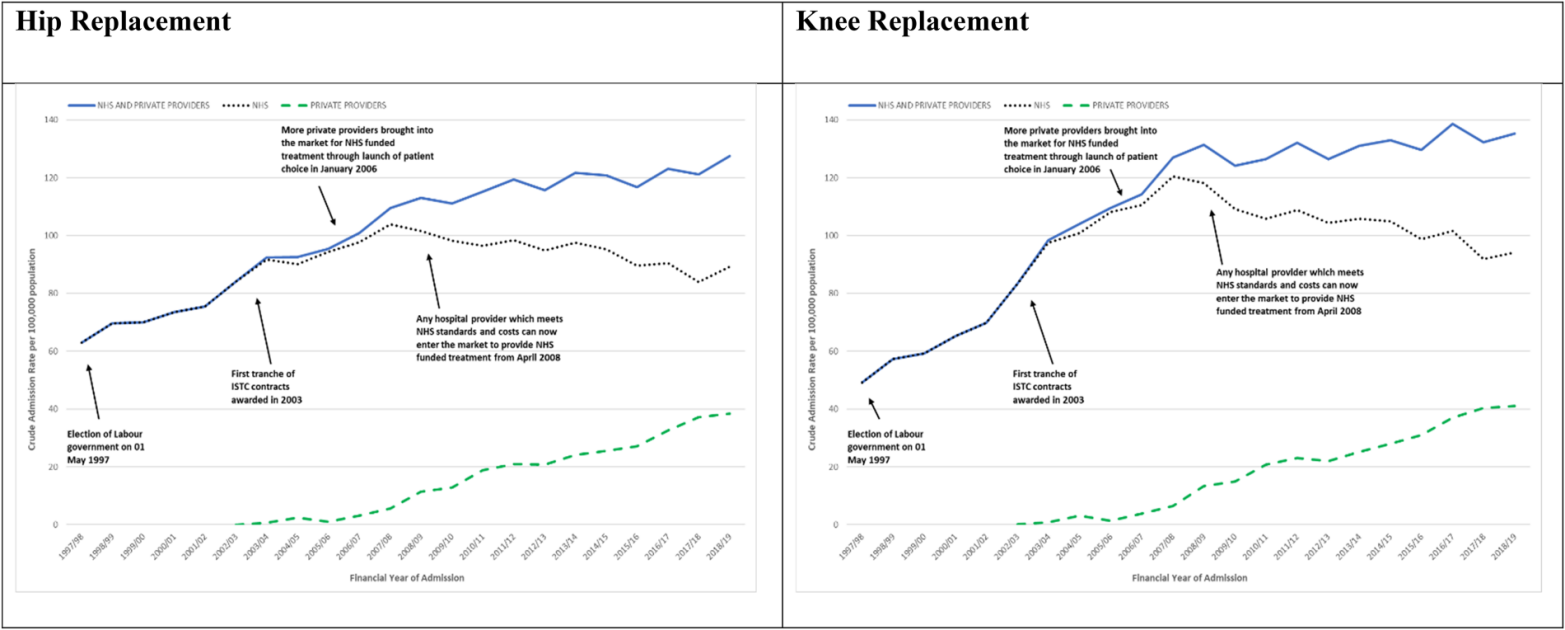
NHS England-funded elective primary hip and knee replacement admissions April 1, 1997, to March 31, 2019. Admission rates overall and for the NHS and private providers.

Between 1997-1998 and 2018-2019 the overall admission rate more than doubled from 62.9 to 127.5 per 100,000; the admission rate to the NHS increased by 41.7 percent from 62.9 to 89.1 per 100,000; and the admission rate to private providers increased from 0 to 38.4 per 100,000 (see Figure 1 and Supplemental Table ST3).

Admission rates for all providers combined rose throughout periods 1, 2, 3, and 4, respectively: 71.9, 92.9, 104.1, and 118.8 per 100,000. Admission rates to the NHS rose and then fell in period 4: 71.9, 91.6, 100.1, and 94.0 per 100,000. Admission rates to private providers rose from 1.4 in period 2 to 4.0 and 24.8 per 100,000 in periods 3 and 4, respectively.

Inequality increased for both the NHS and private providers. Compared with the richest 20 percent (IMD5), the odds of admission to the NHS for the poorest 20 percent (IMD1) decreased from 0.77 in period 1 to 0.67, 0.62, and 0.63 in periods 2, 3, and 4, respectively. The odds of admission to private providers decreased from 0.66 in period 2 to 0.39 and 0.34 in periods 3 and 4, respectively (see Supplemental Table ST5).

Compared with patients with no comorbidity, the odds of being admitted to the NHS for patients with comorbidity increased from 0.16 in period 1 to 0.21, 0.27, and 0.50 in periods 2, 3 and 4, respectively, and in private providers went from 0.03 in period 2 to 0.02 and 0.32 in periods 3 and 4, respectively (see Supplemental Table ST5). In period 2 and period 3, respectively, only 2.8 percent and 2.4 percent of patients admitted to private providers had any comorbidity, compared with 14.1 percent and 17.6 percent of patients admitted to the NHS. Only 6 patients with any comorbidity were admitted to private providers in 2005-2006 and 2006-2007 out of a total of 2,168 patients admitted to private providers in these two years (see Supplemental Table ST7 and Figure SF3).

### Hip Replacement Waiting Times

#### Trends

**Figure 2.**
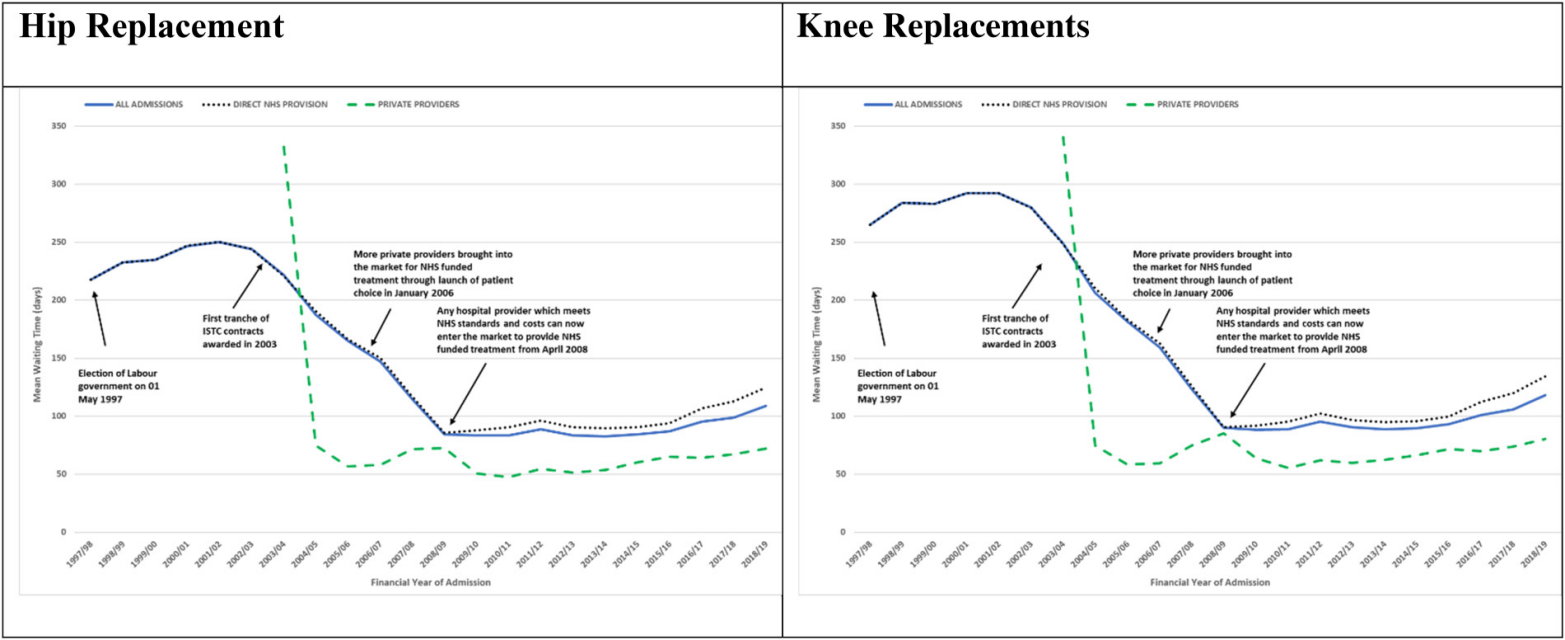
NHS England-funded elective primary hip and knee replacement mean waiting time by financial year. All admissions (all ages) by direct NHS provision and private providers April 1, 1997, to March 31, 2019.

Mean waiting time fell by half between 1997-1998 and 2018-2019, rising from 217.8 days in 1997-1998 to 250.2 days in 2001-2002, then falling to 84.4 days in 2008-2009, plateauing to 84.2 days in 2014-2015 then rising again to 108.9 days in 2018-2019 (see Figure 2 and Supplemental Table ST10).

Mean waiting time fell throughout the periods, from 238.1 in period 1 to 198.6, 132.5, and 89.5 days in periods 2, 3 and 4 overall; from 238.1 days in period 1 to 199.8, 135.1, and 96.9 days in periods 2, 3 and 4 in the NHS; and from 119.2 days in period 2 to 67.1 and 61.4 days in periods 3 and 4 in private providers (see Supplemental Table ST9).

#### Inequality

**Figure 3.**
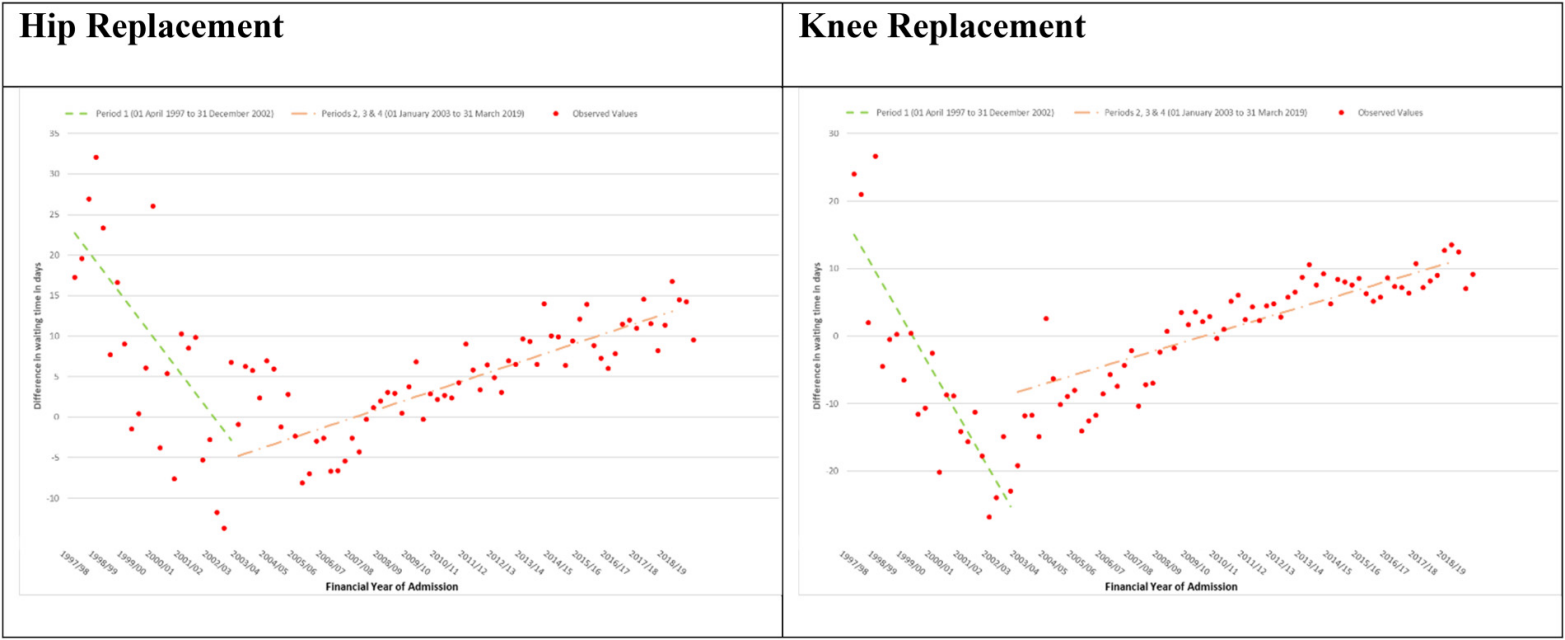
NHS England-funded elective primary hip and knee replacement admissions April 1, 1997, to March 31, 2019. Difference in mean waiting time: most deprived minus least deprived quintile by financial year quarter; modeled using interrupted time series.

Inequality in waiting time between IMD1 and IMD5 decreased −1.16 days per quarter in period 1 (April 1, 1997, to December 31, 2002) and increased 0.29 days per quarter in periods 2, 3 and 4 combined (January 1, 2003, to March 31, 2019) (see Figure 3 and Supplemental Table ST11).

#### The Impact of Private Providers on Waiting Time and Waiting Time Inequality

There were 1,151,006 admissions with waiting time less than three years with complete deprivation quintile and purchase code (see Supplemental Table ST1). Patients admitted to private providers had a mean waiting time almost half that of those admitted to the NHS −49.79 percent (time ratio with 95 percent confidence interval 0.5021 [0.4991, 0.5050]). For every one percentage increase in the admission rate per day, waiting time decreased overall by −1.12 percent (0.9888 [0.9887, 0.9889]). For every one percentage increase in private provider share, waiting time increased overall by 1.93 percent (1.0193 [1.0188, 1.0198]). See Table 2.

**Table 2.** NHS England-Funded Elective Hip Replacement Admissions Basic Model. Waiting Time Ratios with 95% Confidence Intervals and Relative Percentage Change from Loglogistic Accelerated Failure Time Survival Model.

		Time Ratios	Relative Percentage Change
Provider	NHS	1	0
	Private Providers	0.5021 (0.4991, 0.5050)	−49.79%
Admission Rate per Day		0.9888 (0.9887, 0.9889)	−1.12%
Private Provider Share		1.0193 (1.0188, 1.0198)	1.93%
Period 1	Apr 1997-Dec 2002	1	0
Period 2	Jan 2003-Dec 2005	1.0770 (1.0690, 1.0850)	7.70%
Period 3	Jan 2006-Mar 2008	0.8729 (0.8652, 0.8807)	−12.71%
Period 4	Apr 2008-Mar 2019	0.6448 (0.6374, 0.6522)	−35.52%
Area Level Socioeconomic Deprivation	IMD1 (most deprived)	1.0213 (1.0163, 1.0263)	2.13%
	IMD2	1.0127 (1.0083, 1.0170)	1.27%
	IMD3	1.0079 (1.0040, 1.0120)	0.79%
	IMD4	1.0007 (0.9969, 1.0045)	0.07%
	IMD5 (least deprived)	1	0
Comorbidity	No comorbidity	1	0
	One or more comorbidity	0.9995 (0.9965, 1.0024)	−0.05%

Patients in IMD1 had a waiting time 2.13 percent (1.0213 [1.0163, 1.0263]) longer than patients in IMD5 (see Table 2). IMD1 patients admitted to the NHS had a waiting time 1.90 percent (1.0190 [1.0138, 1.0243]) longer than IMD5 patients admitted to the NHS; IMD1 patients admitted to private providers had a waiting time 4.92 percent (1.0492 [1.0333, 1.0655]) longer than IMD5 patients admitted to private providers (see Supplemental Table ST12).

Patients with comorbidity had a waiting time −0.05 percent (0.9995 [0.9965, 1.0024]) shorter than patients without comorbidity although this result was not statistically significant (see Table 2).

### Knee Replacements

#### Admissions

There were 1,246,142 NHS-funded elective primary knee replacement admissions with waiting time less than three years between April 1, 1997, and March 31, 2019 (see Supplemental Table ST1). There were no admissions to private providers in period 1. The number and proportion of admissions to private providers in periods 2, 3, and 4, respectively, were 2,594 (1.68%), 5,414 (3.94%), and 161,296 (20.76%) (see Supplemental Table ST2).

Between 1997-1998 and 2018-2019 the overall admission rate more than doubled from 49.2 to 135.2 per 100,000; the admission rate to the NHS increased by 91.3 percent from 49.2 to 94.2 per 100,000; and the admission rate to private providers increased from 0 to 41.0 per 100,000 (see Figure 1 and Supplemental Table ST4).

Admission rates for all providers combined rose throughout periods 1, 2, 3, and 4 at, respectively, 62.8, 102.7, 119.4, and 131.0 per 100,000. Admission rates to the NHS rose and then fell in period 4: 62.8, 101.0, 114.7, and 103.8 per 100,000. Admission rates to private providers rose from 1.7 in period 2 to 4.7 and 27.2 per 100,000 in periods 3 and 4, respectively.

Inequality increased for both the NHS and private providers. Compared with the richest 20 percent (IMD5), the odds of admission to the NHS for the poorest 20 percent (IMD1) decreased from 0.99 in period 1 to 0.83, 0.77, and 0.85 in periods 2, 3, and 4, respectively. The odds of admission to private providers decreased from 0.72 in period 2 to 0.57 and 0.47 in periods 3 and 4, respectively (see Supplemental Table ST5).

Compared with patients with no comorbidity, the odds of being admitted to the NHS for patients with comorbidity increased from 0.21 in period 1 to 0.27, 0.34, and 0.60 in periods 2, 3 and 4, respectively, and in private providers from 0.05 in period 2 to 0.03 and 0.39 in periods 3 and 4, respectively (see Supplemental Table ST6). In period 2 and period 3, respectively, only 4.6 percent and 3.3 percent of patients admitted to private providers had any comorbidity, compared with 21.2 percent and 25.6 percent of patients admitted to the NHS. Only 8 patients with any comorbidity were admitted to private providers in 2005-2006 and 2006-2007 out of a total of 2,601 patients admitted to private providers (see Supplemental Table ST8 and Figure SF4).

### Knee Replacement Waiting Times

#### Trends

Mean waiting time fell by over half between 1997-1998 and 2018-2019, rising from 265.0 days in 1997-1998 to 292.3 days in 2000-2001, then falling to 90.0 days in 2008-2009, plateauing to 89.5 days in 2014-2015, then rising again to 117.9 days in 2018-2019 (see Figure 2 and Supplemental Table ST10).

Mean waiting time fell throughout the periods, from 283.6 in period 1 to 220.0, 143.1, and 95.7 days in periods 2, 3 and 4 overall; from 283.6 days in period 1 to 221.6, 146.1, and 102.6 days in periods 2, 3 and 4 in the NHS; and from 122.6 days in period 2 to 69.2 and 69.1 days in periods 3 and 4 in private providers (see Supplemental Table ST9).

#### Inequality

Inequality in waiting time between IMD1 and IMD5 decreased −1.83 days per quarter in period 1 (April 1, 1997, to December 31, 2002) and increased 0.32 days per quarter in periods 2, 3 and 4 combined (January 1, 2003, to March 31, 2019) (see Figure 3 and Supplemental Table ST11).

#### The Impact of Private Providers on Waiting Time and Waiting Time Inequality

There were 1,231,854 admissions with waiting time less than three years with complete deprivation quintile and purchase code (see Supplemental Table ST1).

Patients admitted to private providers had a mean waiting time less than half that of those admitted to the NHS −51.72 percent (time ratio with 95 percent confidence interval [0.4828 (0.4801, 0.4855]). For every one percentage increase in the admission rate per day, waiting time decreased overall by −1.01 percent (0.9899 [0.9898, 0.9900]). For every one percentage increase in private provider share, waiting time increased overall by 1.35 percent (1.0135 [1.0131, 1.0140]). See Table 3.

**Table 3.** NHS England-Funded Elective Knee Replacement Admissions Basic Model. Waiting Time Ratios with 95% Confidence Intervals and Relative Percentage Change from Loglogistic Accelerated Failure Time Survival Model.

		Time Ratios	Relative Percentage Change
Provider	NHS	1	0
	Private Providers	0.4828 (0.4801, 0.4855)	−51.72%
Admission Rate per Day		0.9899 (0.9898, 0.9900)	−1.01%
Private Provider Share		1.0135 (1.0131, 1.0140)	1.35%
Period 1	Apr 1997-Dec 2002	1	0
Period 2	Jan 2003-Dec 2005	1.1688 (1.1599, 1.1777)	16.88%
Period 3	Jan 2006-Mar 2008	0.9985 (0.9891, 1.0080)	−0.15%
Period 4	Apr 2008-Mar 2019	0.7123 (0.7042, 0.7205)	−28.77%
Area Level Socioeconomic Deprivation	IMD1 (most deprived)	1.0096 (1.0052, 1.0141)	0.96%
	IMD2	1.0076 (1.0035, 1.0116)	0.76%
	IMD3	1.0047 (1.0009, 1.0085)	0.47%
	IMD4	1.0042 (1.0005, 1.0079)	0.42%
	IMD5 (least deprived)	1	0
Comorbidity	No comorbidity	1	0
	One or more comorbidity	1.0144 (1.0117, 1.0170)	1.44%

Patients in IMD1 had a waiting time 0.96 percent (1.0096 [1.0052, 1.0141]) longer than patients in IMD5 (see Table 3). IMD1 patients admitted to the NHS had a waiting time 0.77 percent (1.0077 [1.0030, 1.0124]) longer than IMD5 patients admitted to the NHS; IMD1 patients admitted to private providers had a waiting time 2.99 percent (1.0299 [1.0158, 1.0442]) longer than IMD5 patients admitted to private providers (see Supplemental Table ST13).

Patients with comorbidity had a waiting time 1.44 percent (1.0144 [1.0117, 1.0170]) longer than patients without comorbidity (see Table 3).

## Discussion

The large increase in the admission rate in the NHS between 1997 and 2003 was associated with a reduction in waiting time inequality for the most deprived 20 percent when the NHS was the sole provider. Following the introduction of private providers in 2003, this trend reversed, overall waiting times increased, and inequalities rose for the poorest 20 percent.

Although admission rates for elective hip and knee surgery more than doubled between 1997 and 2019, the fall in waiting times mainly took place between 2002 and 2008 when admissions to the NHS alone were increasing and the proportion of patients going to private providers was negligible. The introduction of waiting list monies and targets in 2003 allowed hospital consultants to generate additional NHS income on top of their salary for undertaking lists outside of their NHS contracted duties.^28^ It is interesting to note that most of the reduction in waiting times in England took place after the introduction of extra monies for waiting list initiatives. As John Yates has documented, NHS hospital consultants with private practice may have benefited from long waiting lists in the NHS.^29^ After April 2003, with the introduction of private providers in England, hospital consultants continued to be able to supplement their NHS salaries and additional NHS earnings with private practice earnings.

From 2008-2009, waiting times plateaued before rising from 2015-2016 to 2018-2019, coinciding with a decrease in admissions to the NHS and a rise in private sector admissions. Increasing the private sector share of NHS-funded admissions was associated with an increase in waiting times for all patients. For every one percent increase in the share of private sector admissions, waiting times increased by 1.9 percent for hip replacements and 1.4 percent for knee replacements.

The rapid expansion of the private sector has created a two-tier system and was accompanied by a contraction in NHS provision. Not only are waiting times for the private sector half those of the NHS, this two-tier system is driving inequalities in access for the poorest and sickest people. Proportionally fewer patients from the poorest 20 percent are admitted to private providers than to the NHS, and this ratio has worsened over each time period. After 2008, the odds of admission to private providers for the poorest 20 percent compared to the richest 20 percent, and for those with and without comorbidity, were around a half and two thirds, respectively, of that in the NHS. The poorest 20 percent waited around 2 percent and 1 percent longer than the richest 20 percent in the NHS and around 5 percent and 3 percent longer in private providers.

The reason for lower numbers of poorer and less fit patients using the private sector than their wealthier and fitter counterparts is that government policy causes the private sector to treat high volume, low complexity cases. Thus all locally negotiated ISTC contracts for example exclude patients with multiple complex clinical conditions, those at risk of requiring ITU, and those with social issues such as a lack of support on discharge.^30^ The complexity of cases is affected by the lack of intensive care units in most private providers.^31^ In addition, Mason and colleagues found that patients treated by NHS hospitals were more likely to come from income deprived areas than those treated by treatment centers, postulating that patients from areas with higher income deprivation may require more care while in hospital, thereby making it more difficult to discharge them.^30^

Our findings of growing inequality in waiting time for the poorest 20 percent and an increase in overall waiting time associated with private sector use are supported by a number of studies using different methodologies. Cookson and colleagues’ ecological analysis for all treatments found waiting time inequality became increasingly pro-poor until 2004, after which it reversed in favor of the rich.^26^ An international systematic review of orthopedic and cataract surgery found evidence of shorter waiting times for publicly funded patients treated in private providers^32^ as well as evidence that the private sector treated less complex patients.

Kelly and Stoye's econometric analysis of NHS-funded elective hip replacement patients treated in England between April 2002 and March 2013 found the least deprived tercile more likely to be admitted and had significant reduction in their median waiting time compared with the more deprived groups.^19^ Private providers tended to be located in the least deprived areas.^19^

The increased use of the private sector for NHS-funded hip and knee replacements in England is also associated with an increase in overall waiting time. We are unable to ascertain causation in this study, and the direction of this association could be that increasing private sector use pushes up waiting times, or it could be a market response to increasing waiting times by expanding private sector capacity, as the NHS contracts. Other research has favored the former explanation over the latter.

Moscelli and colleagues’ study of market expansion in private provision of NHS-funded treatment showed NHS hospitals with more “rivals” in the area increased overall waiting times at a rate of 5.5 percent for hip replacements and 6.5 percent for knee replacements for each additional provider (NHS or private). No such increase was found for CABG procedures, which are all carried out in the NHS.^11^

Sa and colleagues modeled policies that promote patient choice and increase competition between providers and predicted these policies would have the effect of increasing overall waiting times for cataract surgery. They showed that the imposition of penalties against providers who exceed waiting time targets increased waiting time.^33^

### Limitations

All analysis was on admissions with nonzero waiting time less than three years with valid admission dates. Admission rates are therefore lower than a previous study we published that used all admissions.^34^

The observational nature of this study means causation and temporality could not be determined in the association found between increased private sector use and increasing waiting times.

## Conclusion

Since 2008, NHS capacity in England for hip and knee replacement has fallen sharply and all expansion has been in the private sector. The introduction of private providers into the NHS is associated with increased waiting times for all patients and the development of a two-tier system within the NHS. This two-tier system enables those going to the private sector to have shorter waiting times and hence faster treatment than those going to the NHS. The poorest 20 percent are most affected as they are half as likely to be admitted to the private sector and have longer waiting times than their richer counterparts.

Preferential risk selection by the private sector, reduced in-house NHS provision, and financial pressure from targets may all be contributing to longer waiting times overall and driving inequalities for the poorest 20 percent of patients. Given the general lack of intensive care facilities in private providers, it is inevitable that increasing private sector use will lead to wealthier, healthier, patients using the private sector more than those who are poor and sicker, with the consequent effects we have found on waiting times. There should be a moratorium on private sector use and in-house capacity rebuilt within the NHS to tackle waiting lists and ensure equity.

As a greater share of the budget is spent on outsourcing NHS funded-surgery, further research, a parliamentary committee, and the National Audit Office should examine the costs of outsourcing in England, profits of companies, supplementary income of NHS consultants and associated staff, and the impact of privatizing elective surgery on orthopedic care and other NHS services more widely.

## Supplemental Material

sj-docx-1-joh-10.1177_27551938251336949 - Supplemental material for Outsourcing National Health Service Surgery to the Private Sector: Waiting Time Inequality and the Making of a Two-Tier System for Hip and Knee Replacement in England Supplemental material, sj-docx-1-joh-10.1177_27551938251336949 for Outsourcing National Health Service Surgery to the Private Sector: Waiting Time Inequality and the Making of a Two-Tier System for Hip and Knee Replacement in England by Graham Kirkwood and Allyson M. Pollock in International Journal of Social Determinants of Health and Health Services
