# Supplementary material for: Outsourcing National Health Service Surgery to the Private Sector: Waiting Time Inequality and the Making of a Two-Tier System for Hip and Knee Replacement in England
Source: Int J Soc Determinants Health Health Serv. 2025 Apr 27;55(4):441–50. doi: 10.1177/27551938251336949 (PMC12371136; doi:10.1177/27551938251336949)
Supplement: sj-docx-1-joh-10.1177_27551938251336949 - Supplemental material for Outsourcing National Health Service Surgery to the Private Sector: Waiting Time Inequality and the Making of a Two-Tier System for Hip and Knee Replacement in England [file sj-docx-1-joh-10.1177_27551938251336949.docx]

Supplementary Materials to accompany:

“Outsourcing National Health Service Surgery to the Private Sector: Waiting Time Inequality and the Making of a Two-Tier System for Hip and Knee Replacement in England” by Graham Kirkwood and Allyson M. Pollock

| Table of Contents | Page Number |
| --- | --- |
| 1. Methods | 2 |
| 1.1. Data | 2 |
| 1.2. Measuring deprivation | 2 |
| 1.3. Measuring comorbidity | 3 |
| 1.4. Interrupted time series model building | 3 |
| 1.5. Survival model building | 3 |
| Appendix | 9 |
|  |  |
| 2. Results | 10 |
| 2.1 Figures | 10 |
| 2.2. Admissions, admission rates and odds of admission | 12 |
| 2.3. Waiting times | 21 |
| 2.4. Modelling waiting time | 23 |
| 2.5. Number of sites and private treatment share | 25 |
| References | 29 |

**1. Methods**

**1. 1. Data**

Extracts of secondary care admissions data for NHS-funded elective primary hip and knee replacements were purchased as Hospital Episode Statistics data from NHS Digital at a cost of £11,964. Data were also obtained for cataract surgery, arthroscopies, inguinal hernias, and cholecystectomies; these procedures are not analyzed here.

Hip replacements and knee replacements were requested on the basis of OPCS Classification of Interventions and Procedures (4th edition), OPCS-4, and International Statistical Classification of Diseases and Related Health Problems version 10 (ICD-10) codes in line with the guidance of the Scottish Arthroplasty Project ^1^.

Private providers were identified using HES field PROTYPE set to (IND = Independent sector provider; INDSITE = independent sector provider site; INDSITETC = treatment center at independent sector provider site; INSITE (unclassified); and OTHERPROV = other provider organization). All other provider types in use were classified as the NHS (CARETRUST = care trust; FOUNDATION = NHS foundation trust; PCT = Primary care trust; TRUST = NHS trust; and TRUSTSITETC = treatment center at NHS trust site).

All calculations were by financial year, April 1 to March 31 the following year. Data were analyzed using SAS version 9.4, Stata version 18.0, and Excel.

*Inclusion Criteria*

Episodes were excluded with duplicate HESID and episode key; these tended to be identical apart from the discharge date. Those with the later discharge date were dropped.

Admissions were excluded if ADMIDATE was before ELECDATE or where ELECDATE was set to either 1800-01-01 (“null”) or 1801-01-01 (“invalid date submitted”). Patients were also excluded where their waiting time was three years or more (≥1096 days), the assumption being this was a coding error or at least atypical ^2,3^. Following communication with colleagues at the Freeman hospital in Newcastle upon Tyne, England, admissions with zero days waiting time were also excluded; these tended to be clustered at certain providers suggesting coding issues and possible miscoding of emergency admissions.

For the survival analysis of data, patients were also excluded where: deprivation quintile was missing (as a result of a missing LSOA01 code); or where PURCODE (the organization that commissioned the patient’s health care) was missing.

**1.2. Measuring deprivation**

NHS Digital guidelines were followed, which state that for Index of Multiple Deprivation (IMD) measures, researchers should use “IMD version 2004 on activity up to and including 2006-07; IMD version 2007 on activity between 2007-08 and 2009-10; IMD version 2010 on activity from 2010-11 and M10 2022-23; IMD version 2019 from M11 2022-23.” ^4^

IMD measures by Lower Super Output Area (LSOA), LSOA2001, for 2004, 2007 and 2010 with populations for 2001, 2005 and 2008, were sourced from the Department for Levelling Up, Housing and Communities and Ministry of Housing, Communities & Local Government ^5^. These were used to derive population weighted deprivation measures by quintile for England by LSOA01 with quintile 1 = most deprived and quintile 5 = least deprived. These quintiles provide approximately equal numbers of population in each such that equality implies an equal number of admissions in each quintile for each financial year.

**1.3. Measuring comorbidity**

Comorbidity was determined by adapting the method outlined by the Royal College of Surgeons. ^6^ Due to the absence of prior admissions for all causes, acute conditions in the previous year could not be included for myocardial infarction (ICD10 codes: I21, I22, I23), chronic pulmonary disease (J46) and renal disease (N171, N172, N19).

**1.4. Interrupted time series model building**

The level and trend change model described by Wagner et al was used ^7^. For each of the procedures, the full model including time, level and trend changes was fitted and compared, using an F test, to the reduced model containing only level change terms. ^8^ Autocorrelation was checked for using the Durbin-Watson test and terms included at a significance level of *p* < 0.1 and where necessary adjusted for up to and including a lag level of five to include the possibility of seasonality. ^9^ Heteroscedasticity was also checked for at the level of *p* < 0.1 and where necessary adjusted for.

The model was constructed as:

T = time in yearly quarters (1, 2, …, 88), LC = level change and SC = slope (trend) change

Difference in Mean Waiting Time = a_0_ + a_1_T + a_2_LC_1_ + a_3_SC_1_

period one (April 1, 1997 to December 31, 2002): T = 1, …, 23; LC_1_ = 0; SC_1_ = 0

period two (January 1, 2003 to March 31, 2019): T = 24, …, 88; LC_1_ = 1; SC_1_ = T – 23

Autoregressive integrated moving average (ARIMA) models were checked against autoregressive models (AUTOREG) using the AIC (Akaike information criterion) measure as a judgement of fit. The SAS procedure AUTOREG was chosen in preference to ARIMA as a result of lower AIC measures (table SM1). For both treatments the full model including time, level and trend change terms fitted the data better than the reduced model with level change terms only (both *p* < 0.0001). An adjustment was made for autocorrelation at the first level for hip replacements and third level for knee replacements. Both models were adjusted for heteroscedasticity.

**Table SM1. NHS England Funded Elective Primary Hip and Knee Replacement Admissions April 1, 1997 to March 31, 2019. Interrupted Time Series Comparison of Best Model, Arima vs. Autoreg, Akaike Information Criterion (AIC)**

|  | **ARIMA** | **AUTOREG** |
| --- | --- | --- |
| Hip replacement | 565.6 | 516.7 |
| Knee replacement | 550.3 | 509.6 |

**1.5. Survival model building**

The assumption of proportional hazards between the categorical variables deprivation, provider type and comorbidity was tested visually by plotting log cumulative hazards [ln(-ln $\hat{S}$(t) )] (see Appendix) against the log of time measured in days waited using data for period 4 (01 April 2008 – 31 March 2019) when the private sector was most active. To uphold the assumption of proportional hazard, the lines for each category within a variable should be parallel.

For both treatments, the log cumulative hazard plot has noticeable divergence for deprivation and comorbidity at shorter waiting times (figure SM1a & SM1b). There is clear divergence and a crossing of lines for the plots for NHS and private providers. Therefore, the life table estimates of the hazard function were plotted by period to allow assessment of a suitable parametric form for the hazard function.

Within each period, the graphs of the life table estimate of the hazard function were approximately unimodular, apart from period 2, the shortest time period. This combined with the fact that hazard rates increase then decrease suggested that the parametric accelerated failure time lognormal and loglogistic models might be reasonable approximations (figure SM2a, SM2b). ^10^ The mode of each graph varied by period, likely due to differing waiting time targets.

Models were built and tested for shared frailty with all variables, on the fields: PURCODE (organization commissioning patient's health care); CCG_GP_PRACTICE (CCG of patient’s GP); CCG_RESIDENCE (CCG of patient’s home); LSOA01 (2001 census Lower Layer Super Output Area); LSOA11 (2011 census Lower Layer Super Output Area); and REFERORG (code of the organization from which the referral was made, such as GP practice or NHS trust). Data were used where each of these fields was non missing (hip replacements N = 555,952; knee replacements N = 610,873) which meant data were only from 2009/10 (with one admission in 2008/09 for knee replacements). AIC measures were used to select the most parsimonious model. The loglogistic model with shared frailty on PURCODE was chosen as it provided the lowest AIC measure for both treatments (table SM2).

**Table SM2. NHS England Funded Elective Hip and Knee Replacement Admissions * (Hips, N= 555,952; Knees N=610,873). Akaike Information Criterion (AIC) to Assess Goodness of Fit of Survival Models on Waiting Time Comparing Shared Frailty terms (Frailty Distribution = Gamma)**

| **Shared Frailty Term** | **Hip Replacement** |  | **Knee Replacement** |  |
| --- | --- | --- | --- | --- |
|  | **Lognormal** | **Loglogistic** | **Lognormal** | **Loglogistic** |
| **CCG_GP_PRACTICE** | 1,366,470 | 1,342,003 | 1,489,699 | 1,460,865 |
| **CCG_RESIDENCE** | 1,366,442 | 1,342,177 | 1,489,666 | 1,460,966 |
| **LSOA01** | 1,440,194 | 1,392,476 | 1,561,243 | 1,508,980 |
| **LSOA11** | 1,440,202 | 1,392,488 | 1,561,255 | 1,508,993 |
| **REFERORG** | 1,421,164 | 1,362,905 | 1,539,676 | 1,478,629 |
| **PURCODE** | 1,351,572 | 1,322,008 | 1,473,424 | 1,440,273 |

* - with non-missing CCG_GP_PRACTICE, CCG_RESIDENCE, LSOA01, LSOA11, REFERORG and PURCODE fields

The two “external variables:” number of admissions per day during a patient’s wait; and the share of admissions to private providers during a patient’s wait; are of the type Collett describes as “one that exists totally independently of any particular individual, such as the level of atmospheric sulphur dioxide, or air temperature. Changes in the values of such quantities may well have an effect on the lifetime of individuals” ^10^ ^(p. 296)^. Our measures of the level of volume of admissions and private sector share in the “environment” while a patient waited for their NHS funded hip or knee replacement are analogous to these atmospheric variables and may well have an effect on a patient’s waiting time.

Adding an interaction between provider and deprivation significantly improved the model for both treatments (likelihood ratio test: hip replacements *p* < 0.0001; knee replacements *p* = 0.0010).

**Figure SM1a. Log cumulative hazards against log of time – Hip replacements**

| DEPRIVATION | COMORBIDITY |
| --- | --- |
| 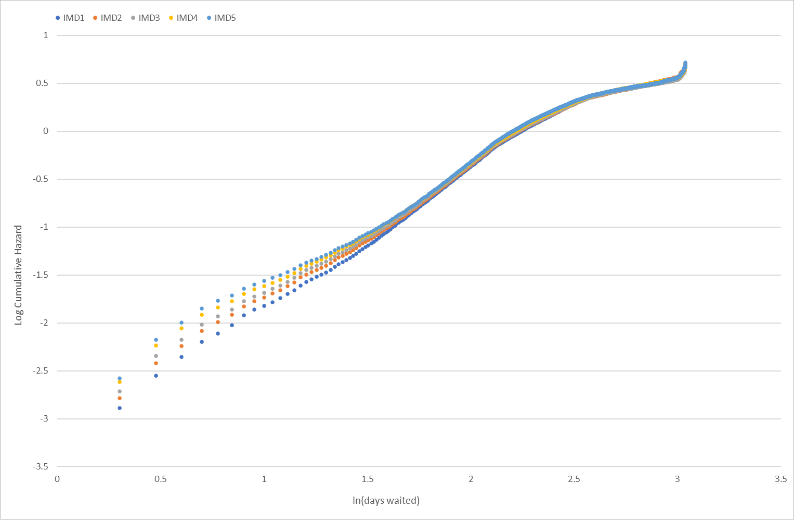 | 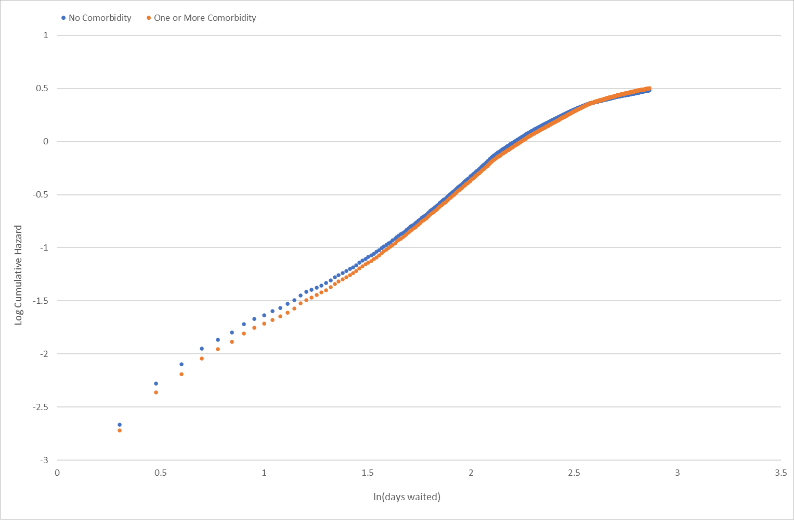 |
|  |  |
| NHS OR PRIVATE PROVIDER |  |
| 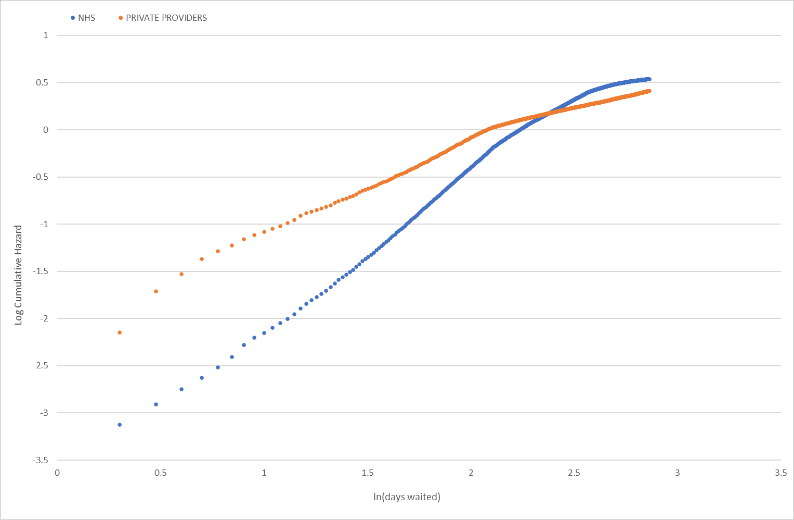 |  |

**Figure SM1b. Log cumulative hazards against log of time – Knee replacements**

| DEPRIVATION | COMORBIDITY |
| --- | --- |
| 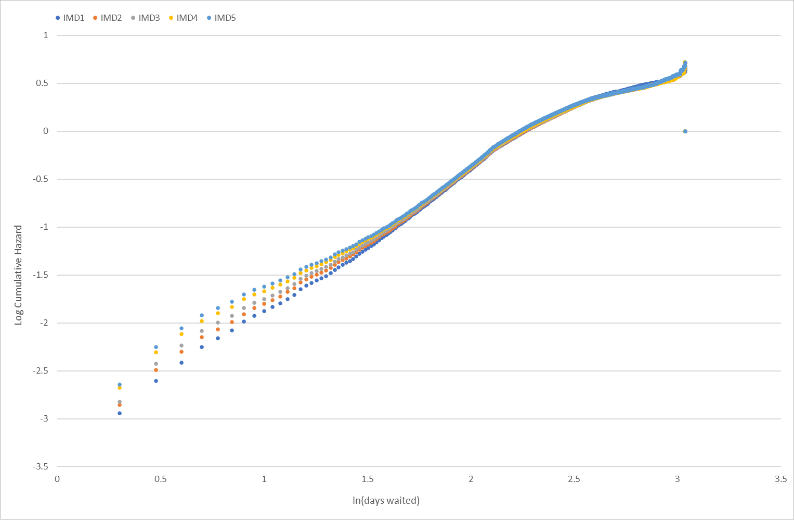 | 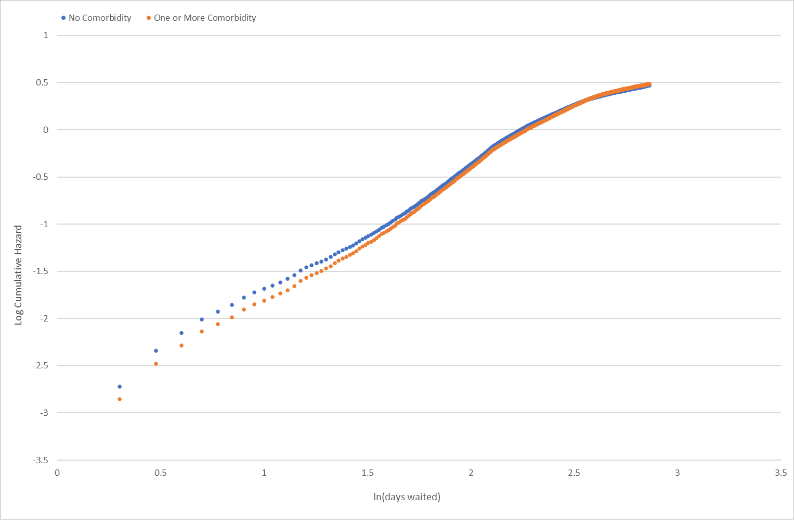 |
|  |  |
| NHS OR PRIVATE PROVIDER |  |
| 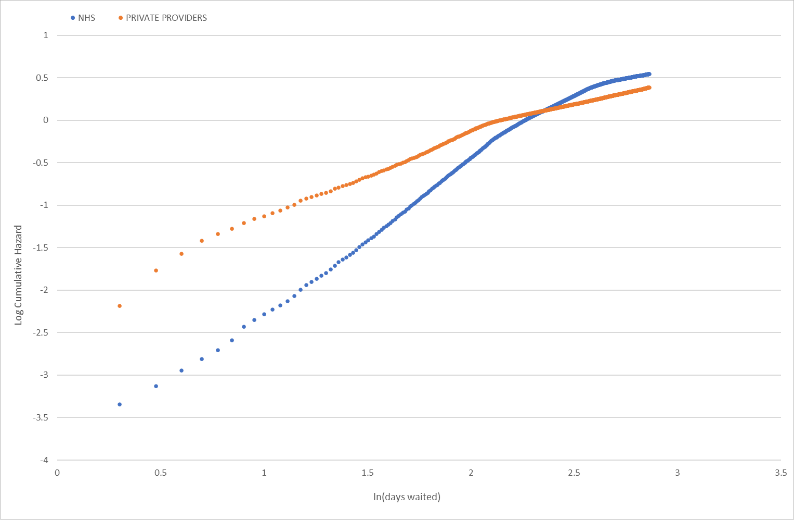 |  |

**Figure SM2a. NHS England Funded Elective Hip Replacement Admissions 01 April 1997 to 31 March 2019. Life table estimates of the hazard function P (T=t | T>=t) in weeks by period**

| **Period 1 (01 April 1997 – 31 December 2002)** | **Period 2 (01 January 2003 – 31 December 2005)** |
| --- | --- |
| **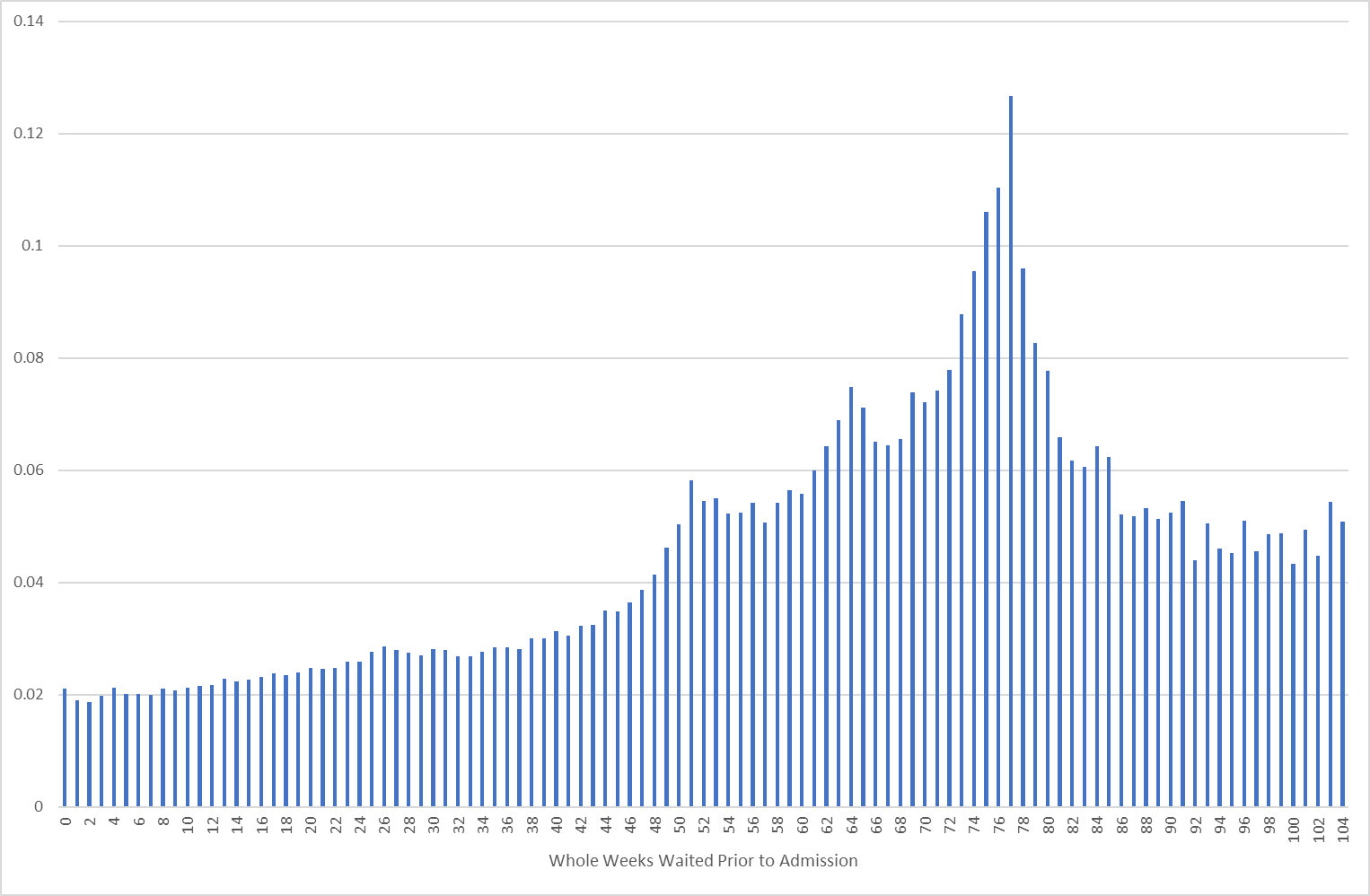** | **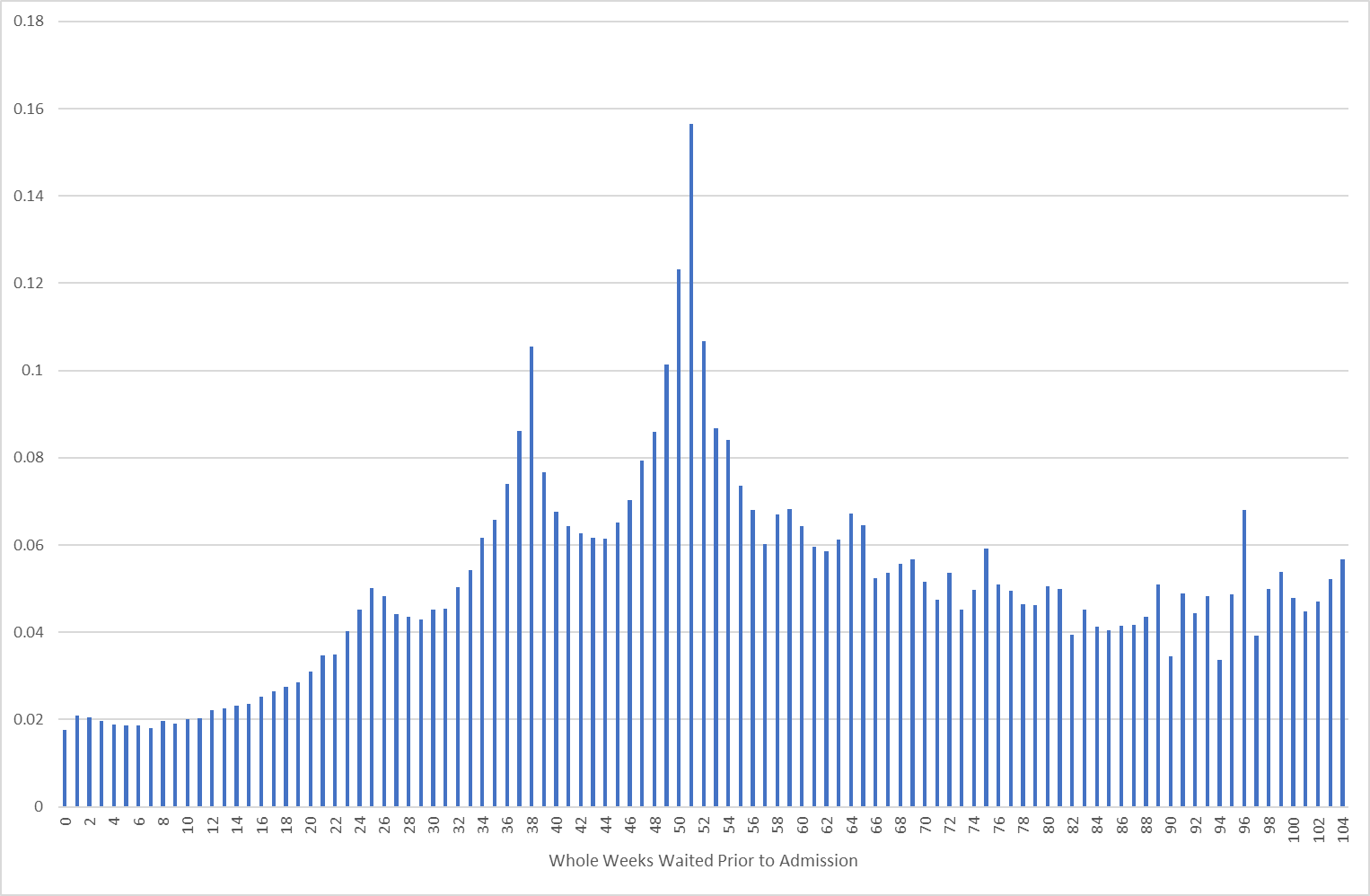** |
| **Period 3 (01 January 2006 – 31 March 2008)** | **Period 4 (01 April 2008 – 31 March 2019)** |
| **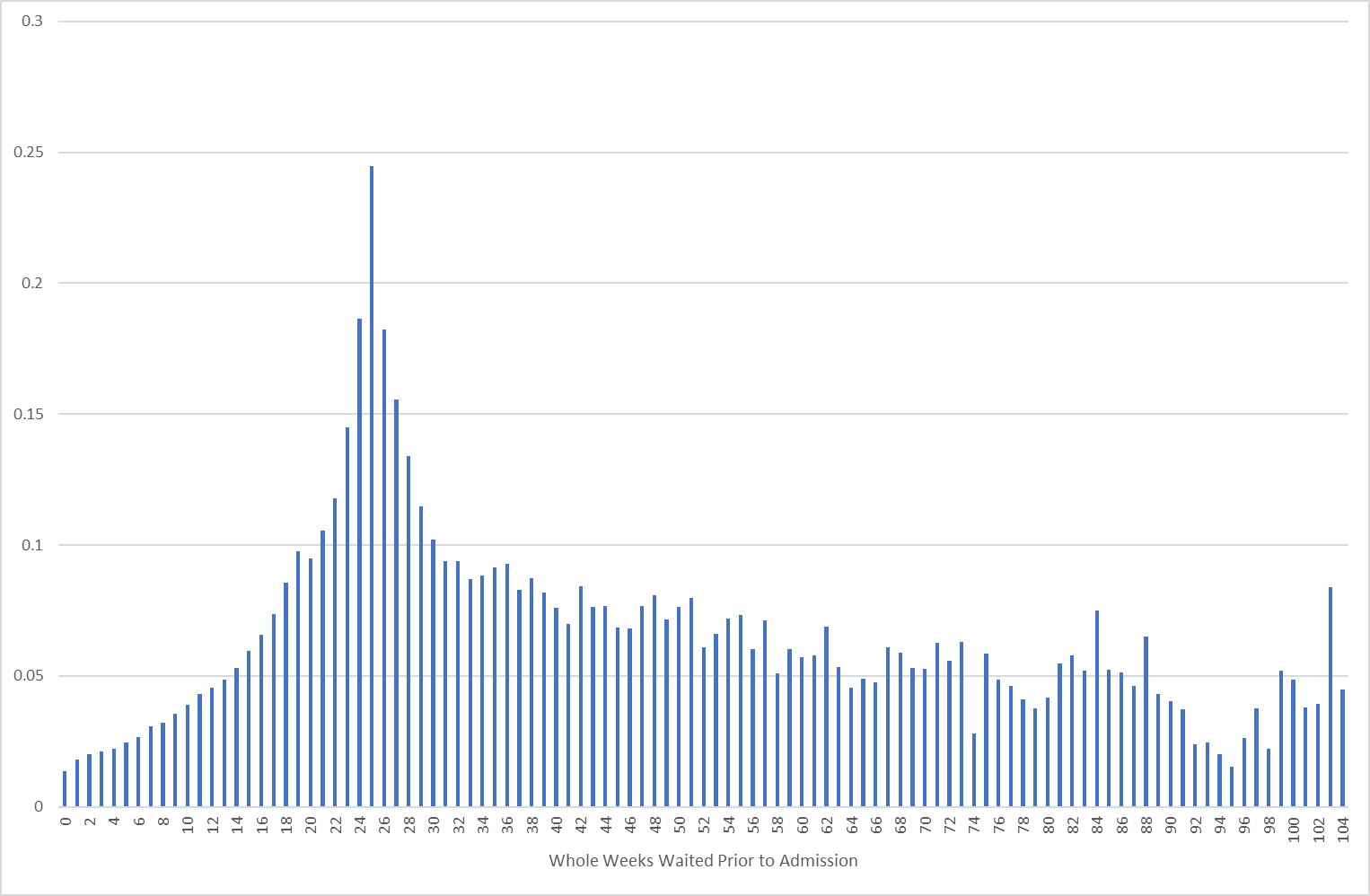** | **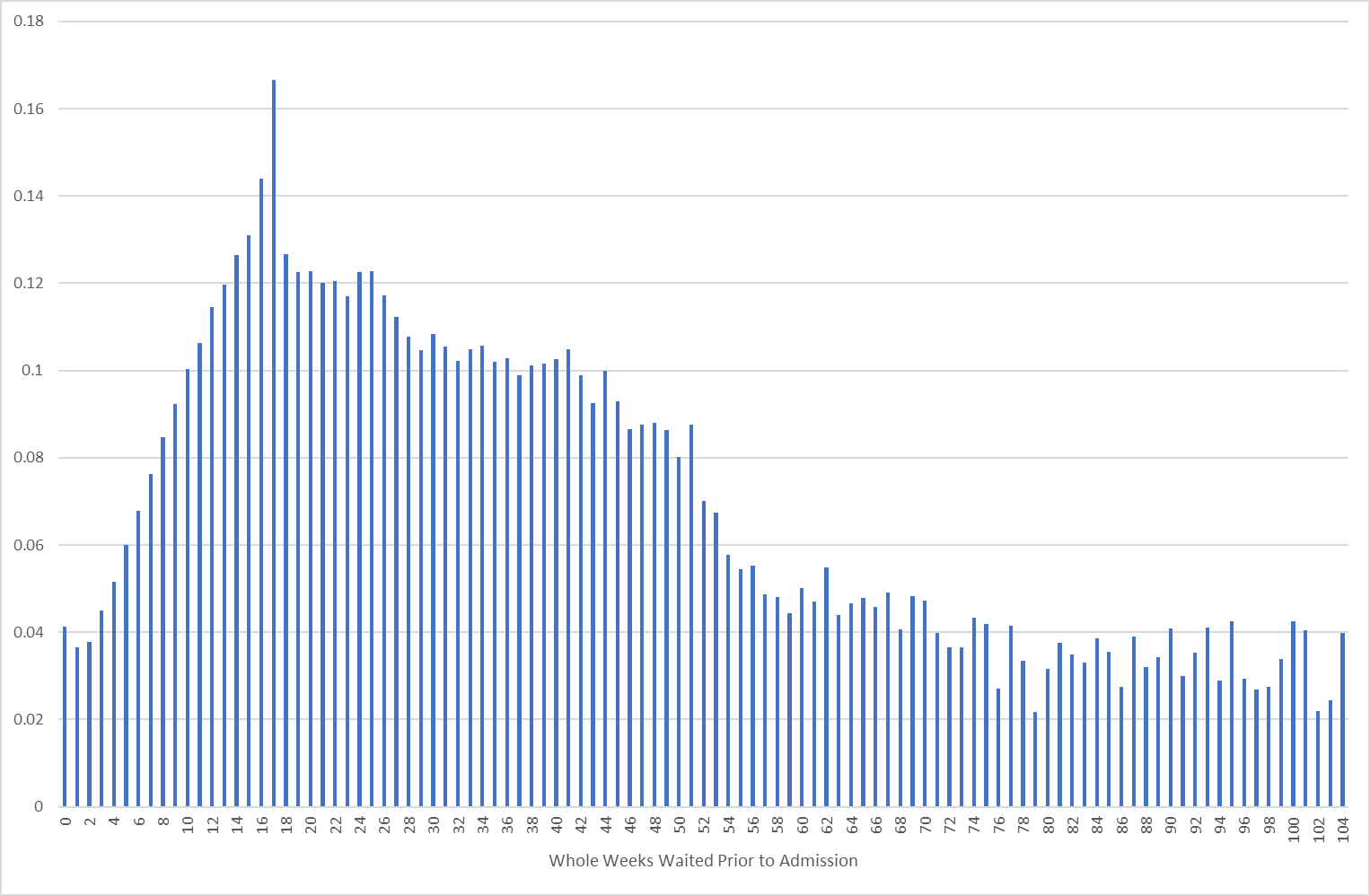** |

**Figure SM2b. NHS England Funded Elective Knee Replacement Admissions 01 April 1997 to 31 March 2019. Life table estimates of the hazard function P (T=t | T>=t) in weeks by period**

| **Period 1 (01 April 1997 – 31 December 2002)** | **Period 2 (01 January 2003 – 31 December 2005)** |
| --- | --- |
| **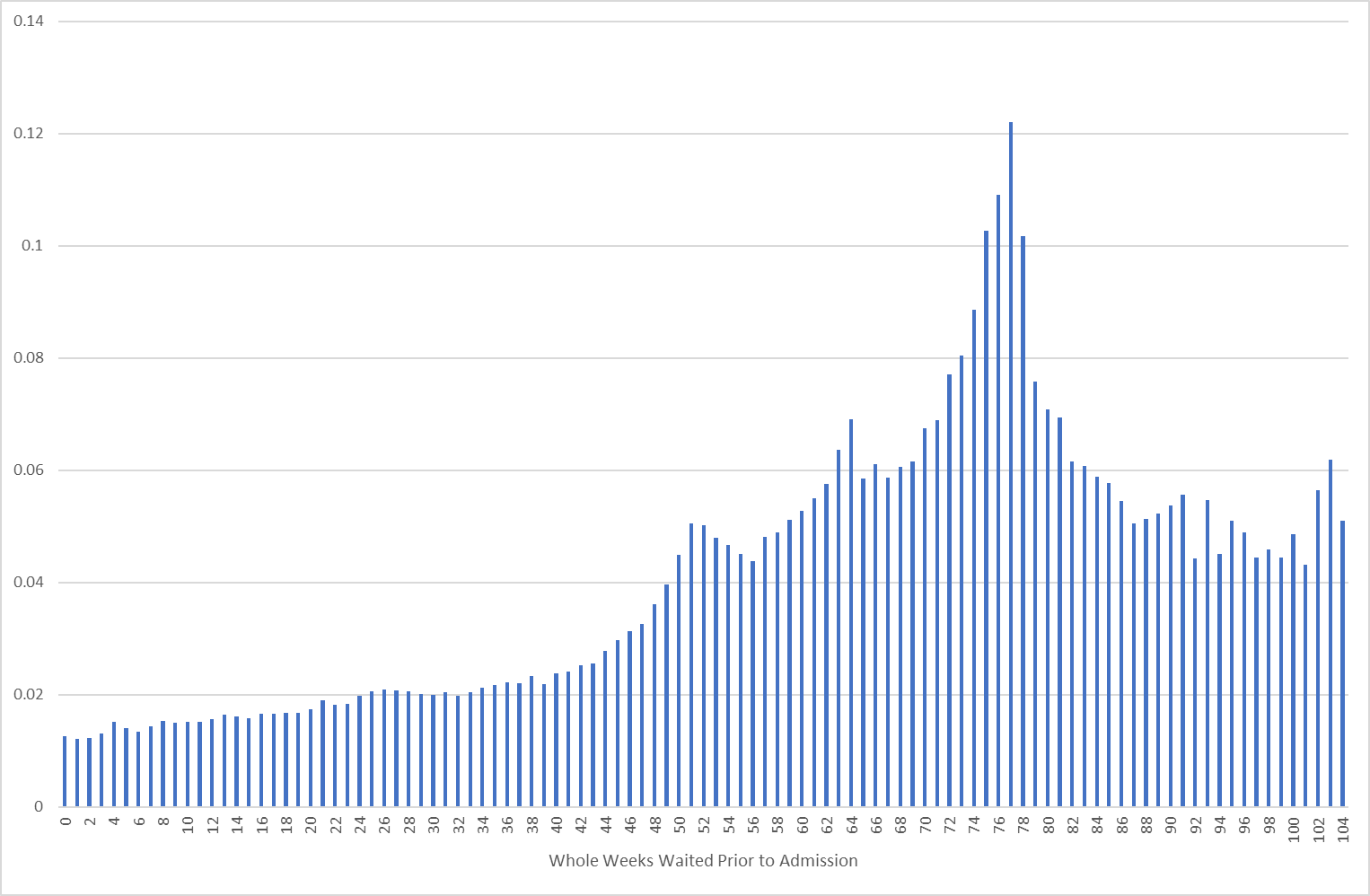** | **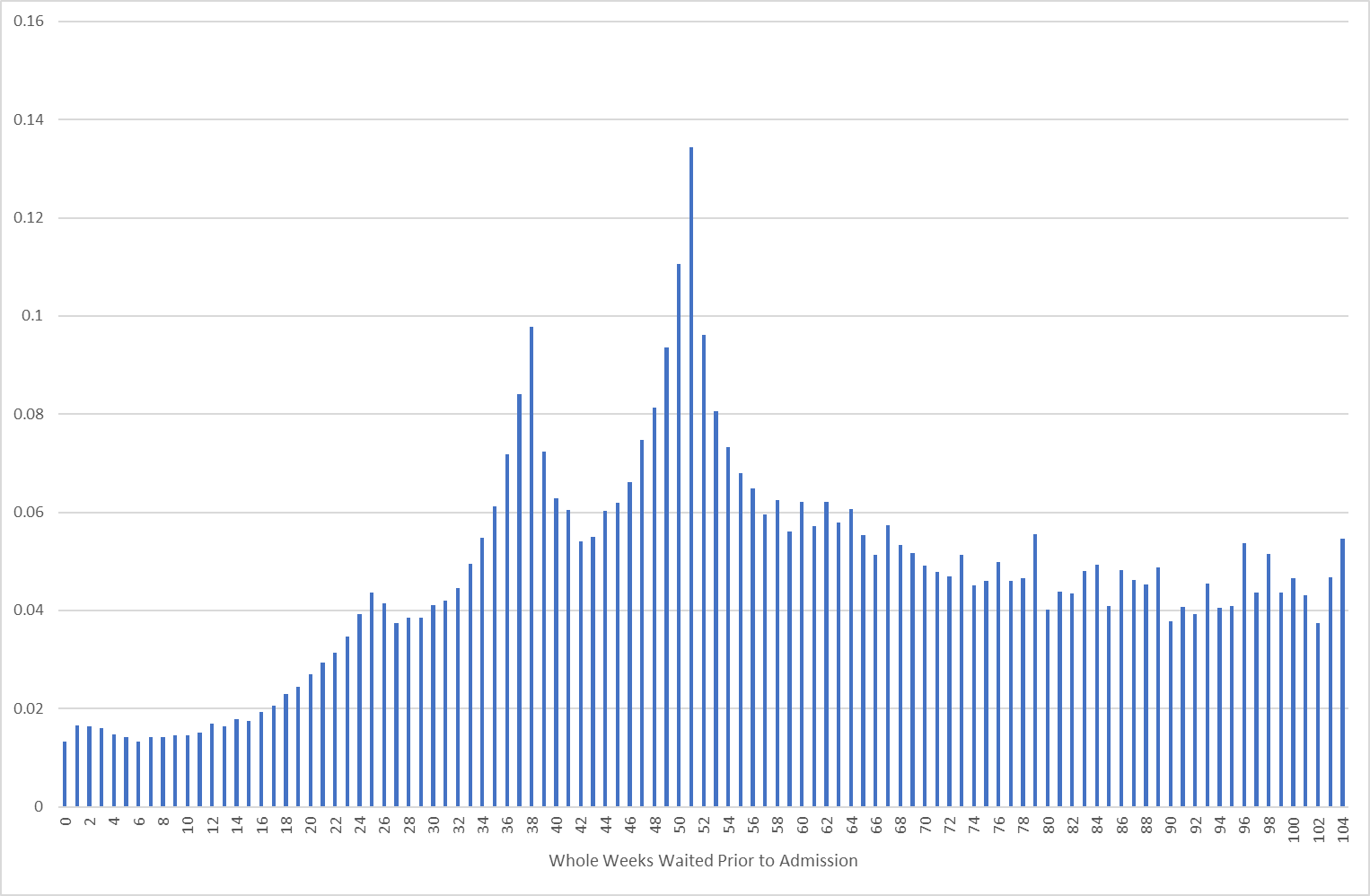** |
| **Period 3 (01 January 2006 – 31 March 2008)** | **Period 4 (01 April 2008 – 31 March 2019)** |
| **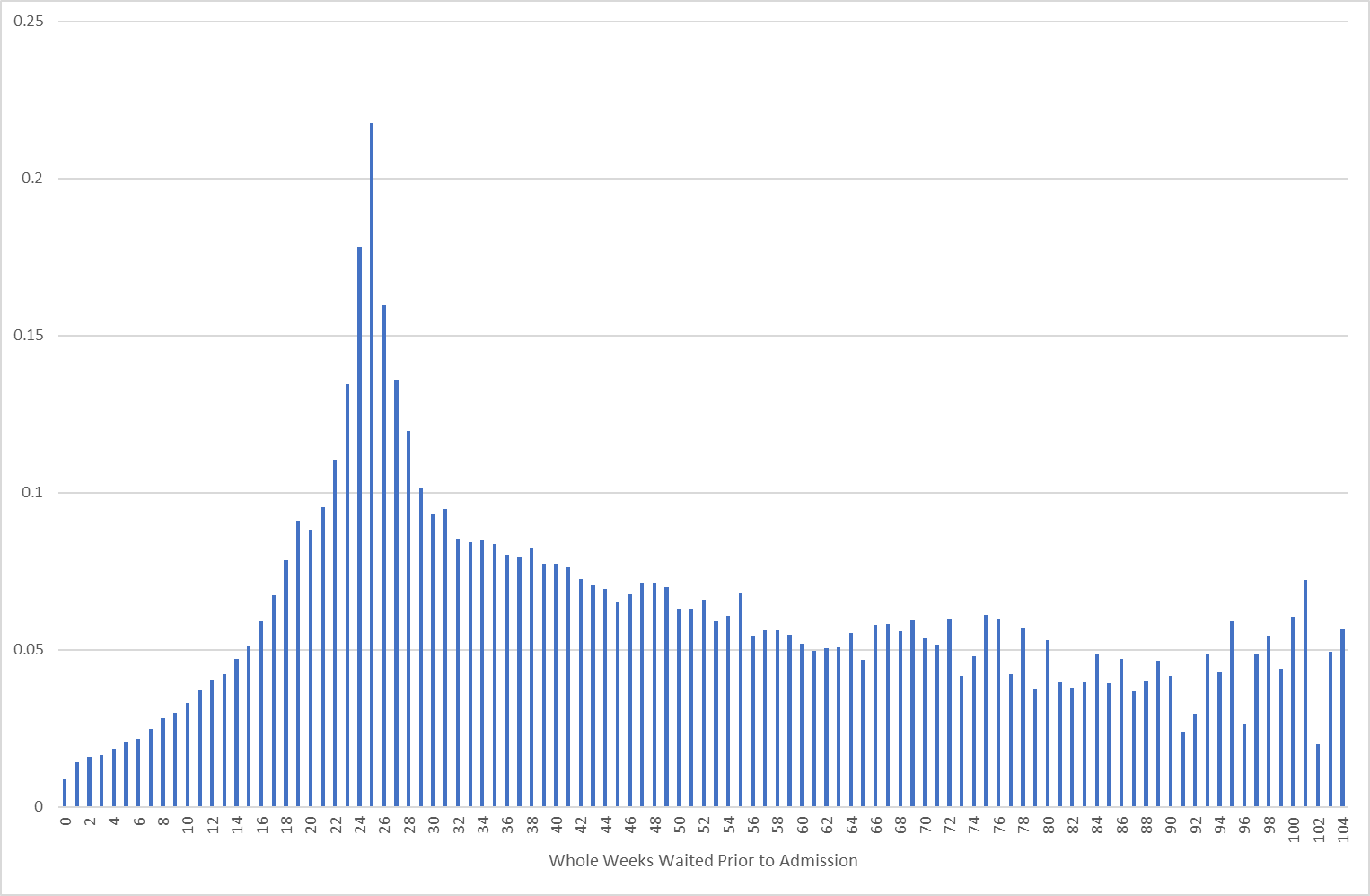** | **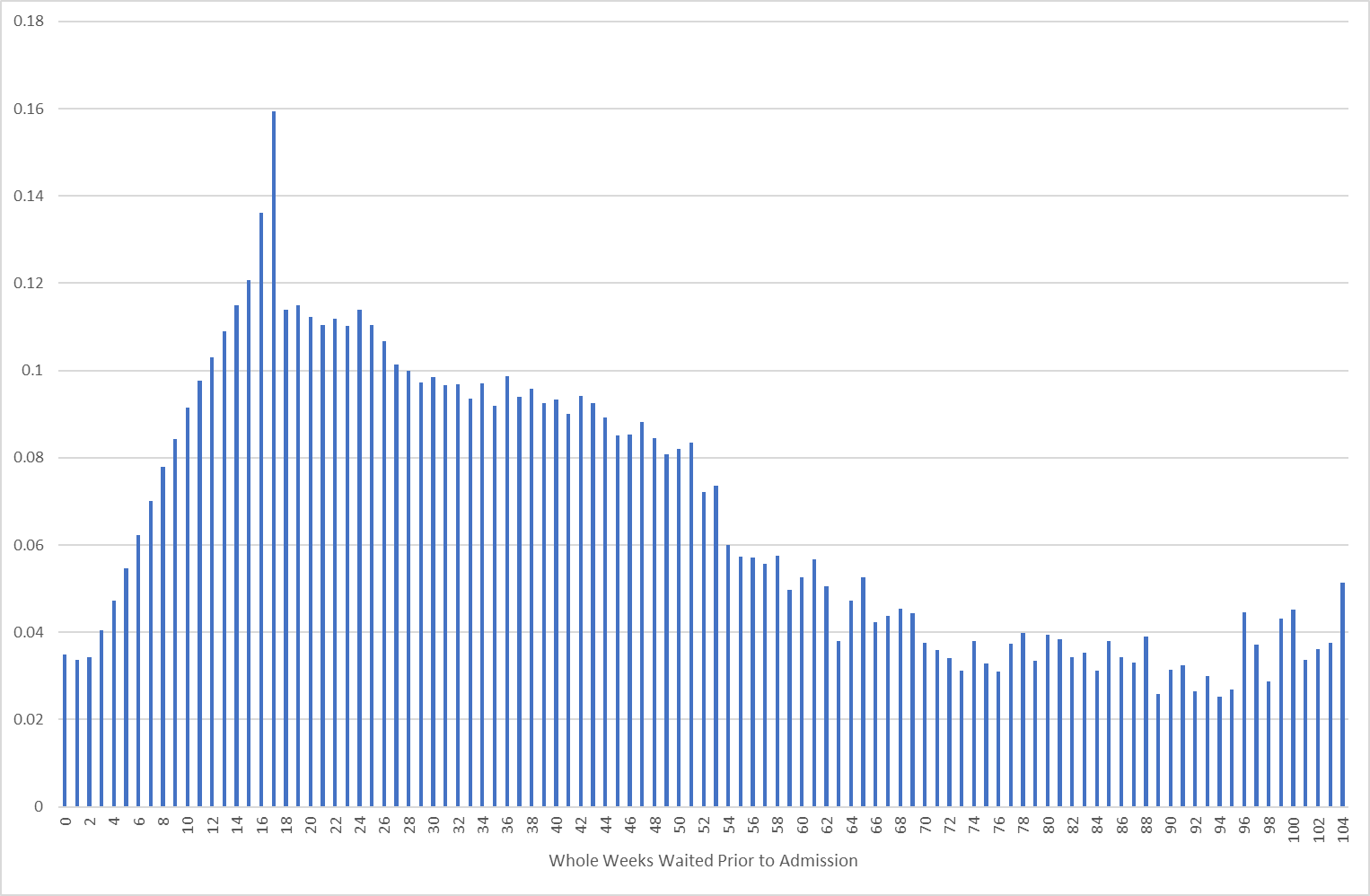** |

**Appendix**

**Survivor Function**

The empirical survivor function ^10^ ^(p. 17)^ is defined as:

$\hat{S}$(t) = Number of individuals with survival times ≥ t / Number of individuals in the data set

This translates to:

$\hat{S}$(t) = Number of patients still on waiting list at time t / Total number of patients admitted overall

**Hazard function**

The hazard function h(t) is the instantaneous probability of admission at time t. Given that all intervals for NHS admission data is in equal amounts of one day, the **life table estimate of the hazard function ^10^ ^(p. 31)^** can be calculated as:

h*(t) = number of admissions on day t / (number of patients still on waiting list on day t minus half the number of admissions on day t)

**2. RESULTS**

**2.1 Figures**

**Figure SF1. NHS England Funded Elective Primary Hip and Knee Replacement Admissions 01 April 1997 to 31 March 2019. Number of Private Provider Sites by Financial Year Quarter**

| **Hip Replacement** | **Knee Replacement** |
| --- | --- |
| **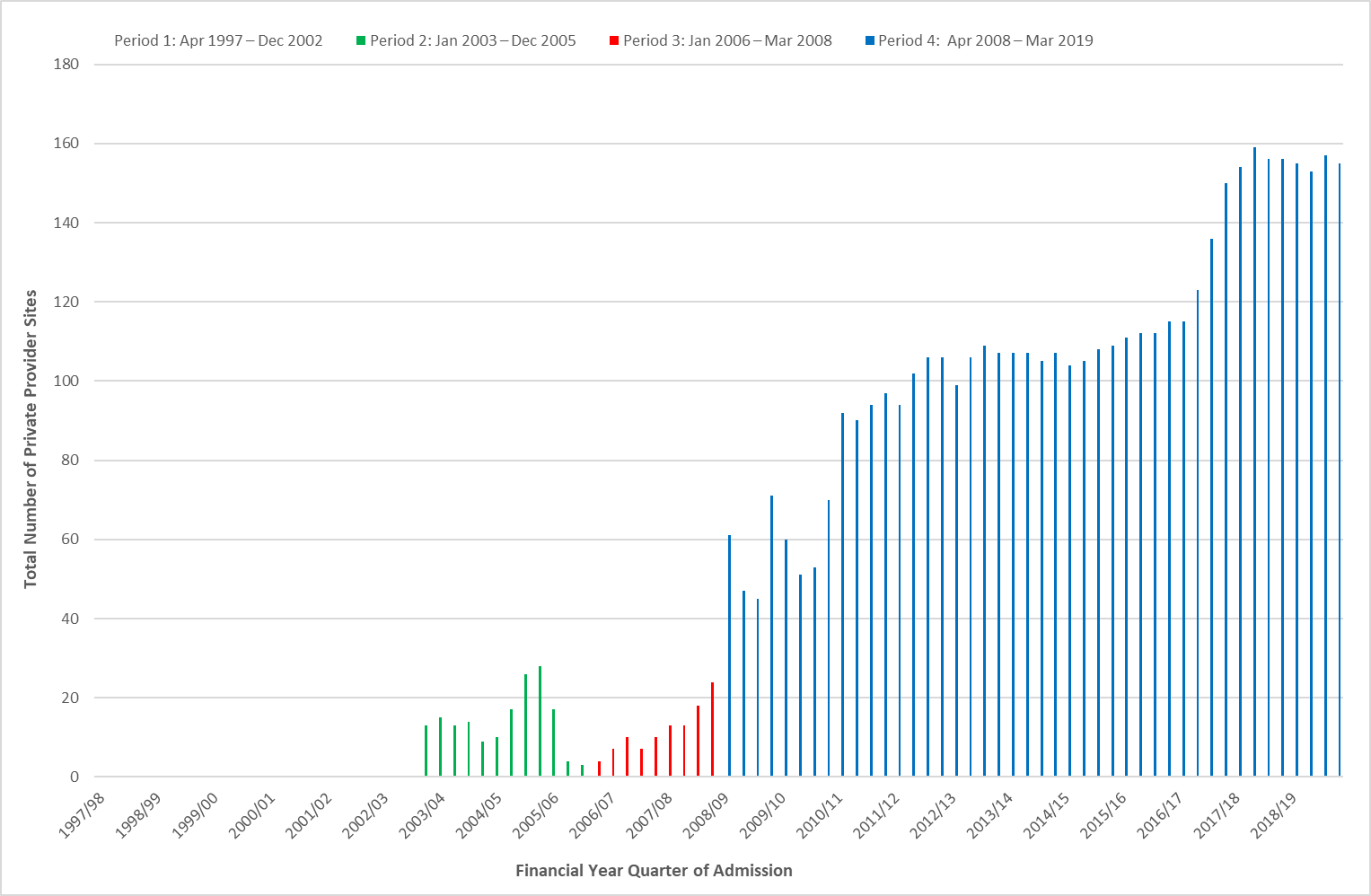** | **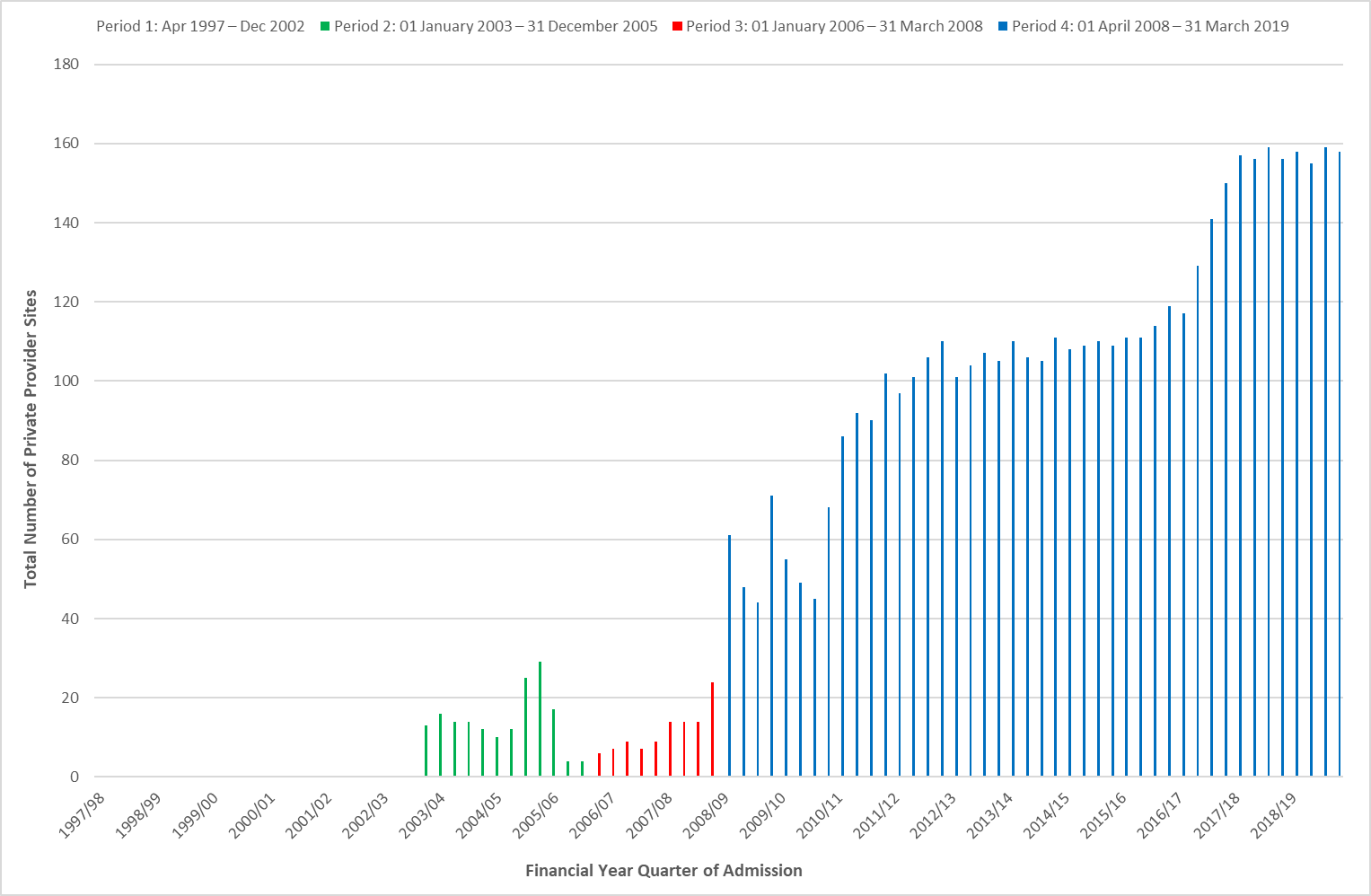** |

**Figure SF2. NHS England Funded Elective Primary Hip and Knee Replacement Admissions 01 April 1997 to 31 March 2019. Share of Admissions to Private Providers by Financial Year Quarter**

| **Hip Replacement** | **Knee Replacement** |
| --- | --- |
| **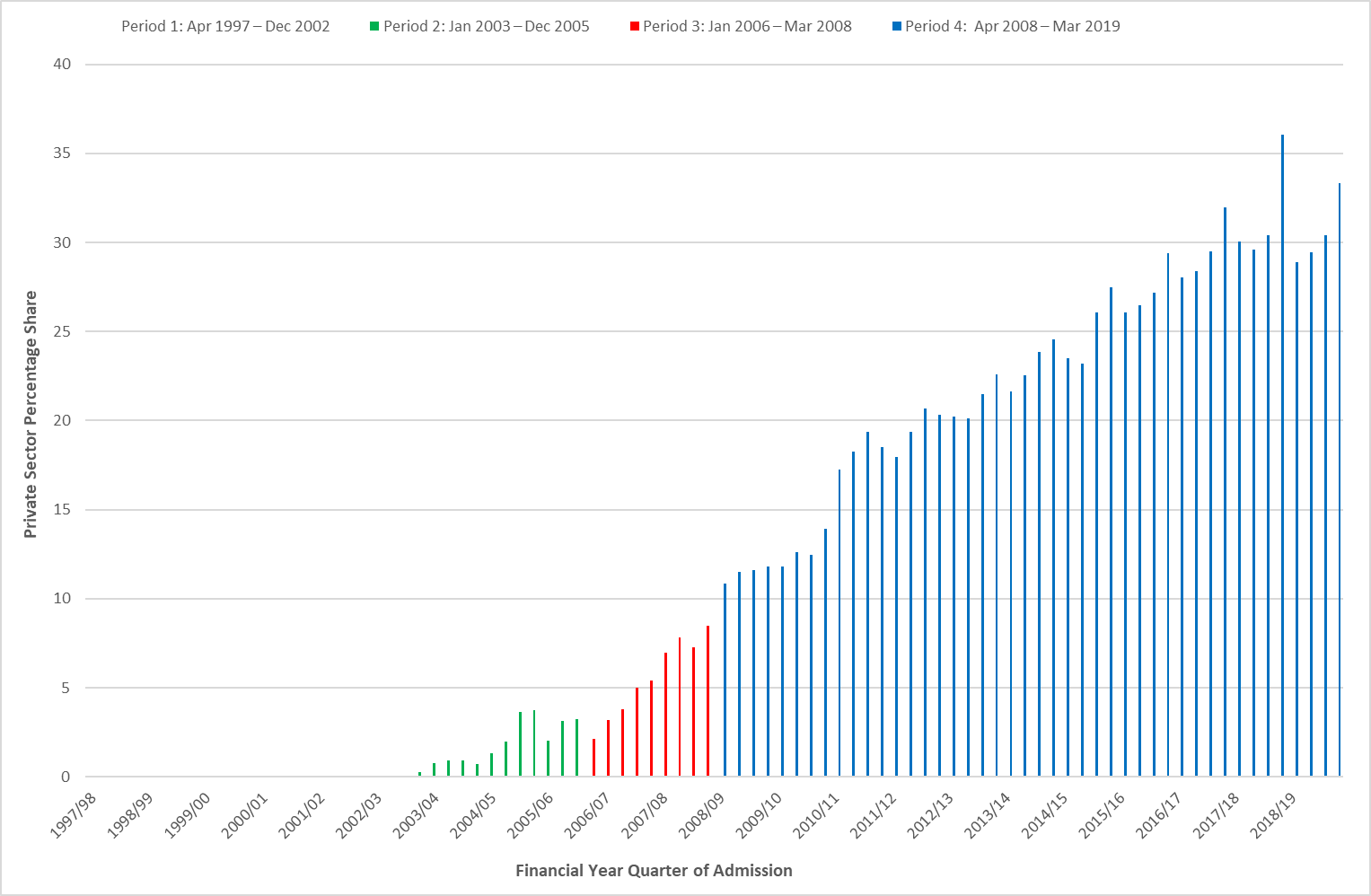** | 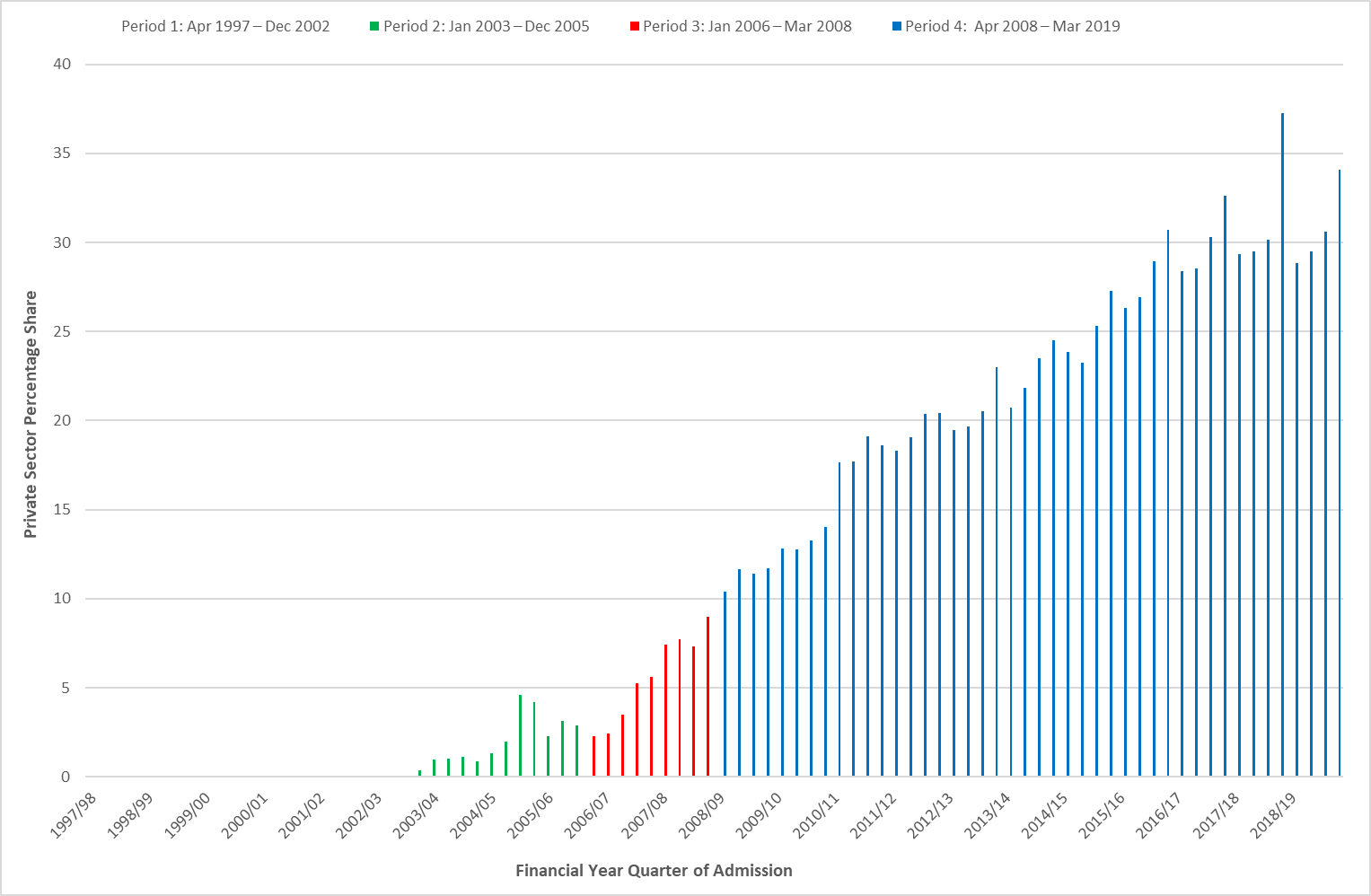 |

**Figure SF3. NHS England Funded Elective Primary Hip Replacement Admissions 01 April 1997 to 31 March 2019. Odds of Admission by Deprivation and Comorbidity by Financial Year**

| **IMD** | **COMORBIDITY** |
| --- | --- |
| **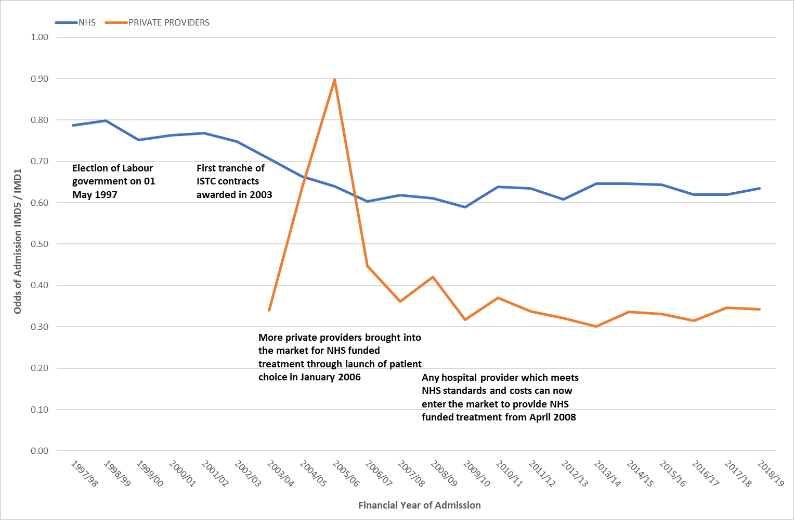** | **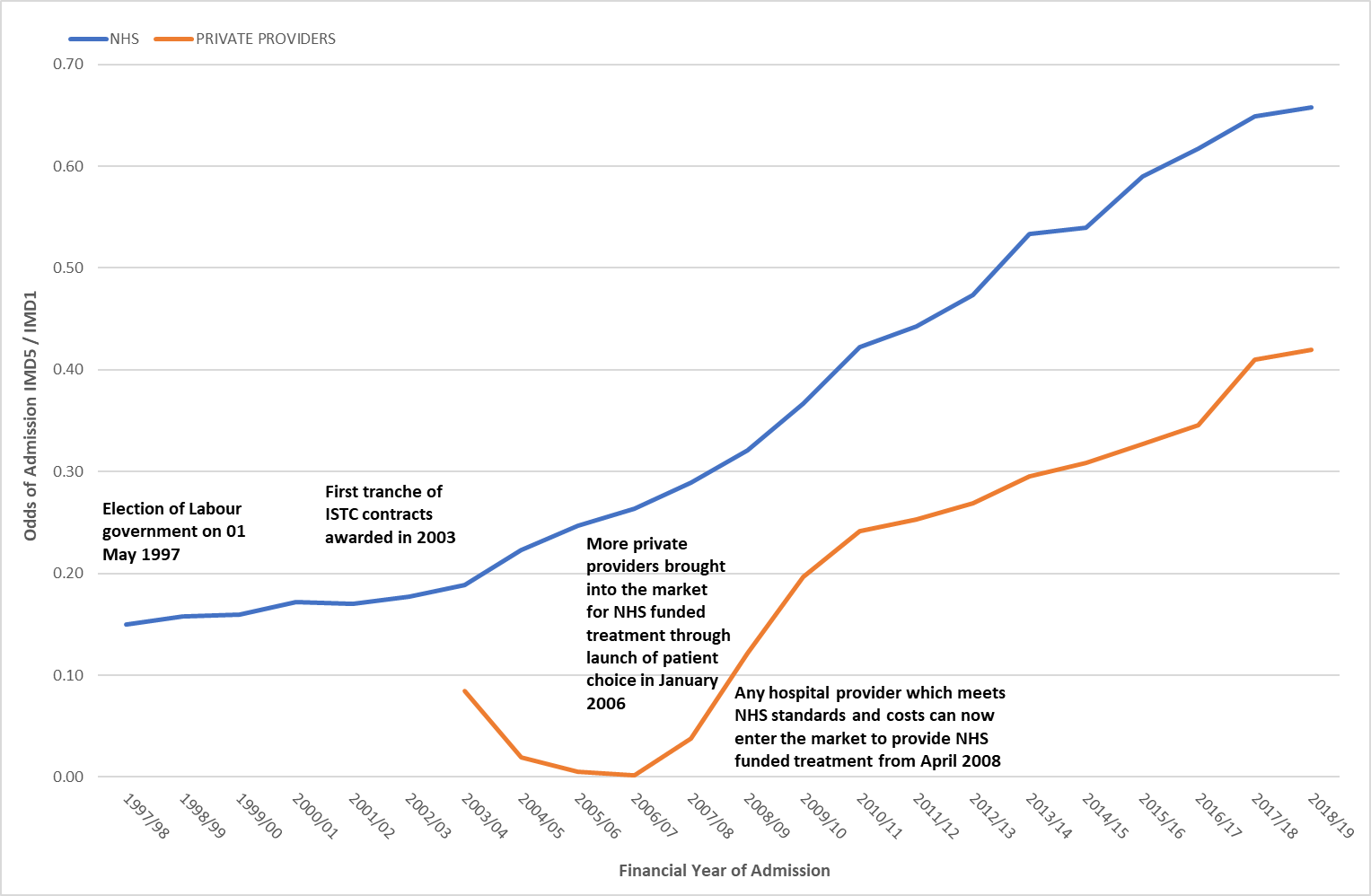** |

**Figure SF4. NHS England Funded Elective Primary Knee Replacement Admissions 01 April 1997 to 31 March 2019. Odds of Admission by Deprivation and Comorbidity by Financial Year**

| **IMD** | **COMORBIDITY** |
| --- | --- |
| **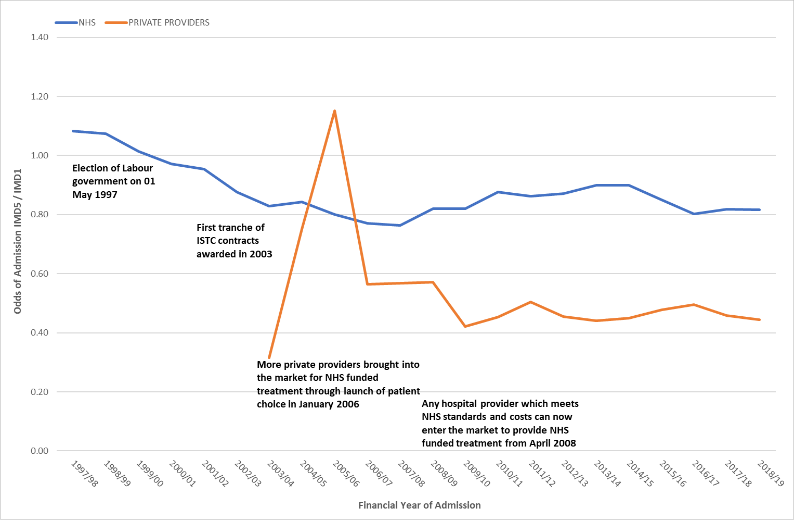** | **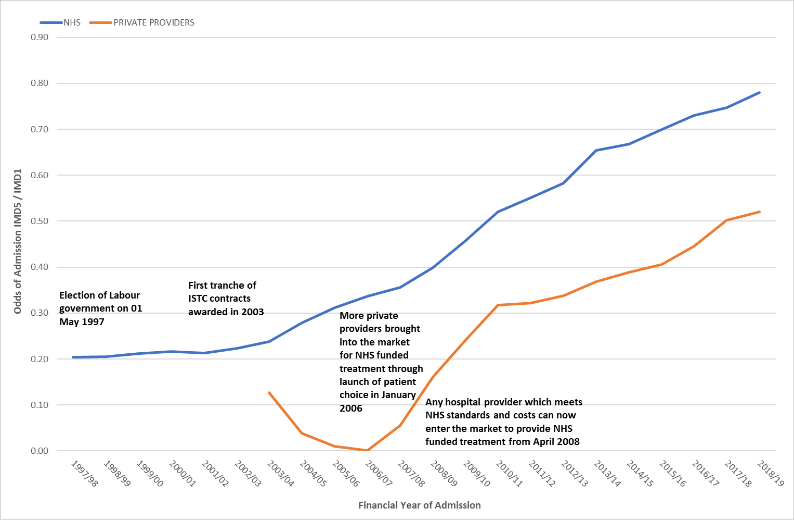** |

**2.2. Admissions, admission rates and odds of admission**

**Table ST1. Details of datasets under inclusion criteria**

|  | **Hip Replacement** | **Knee Replacement** |
| --- | --- | --- |
| **Admissions between 01 April 1997 and 31 March 2019** | 1,243,578 | 1,326,991 |
| **Minus duplicates on ENCRYPTED_HESID EPIKEY** | 1,241,878 | 1,324,994 |
| **ELECDATE ≤ ADMIDATE and ELECDATE not in (1800-01-01, 1801-01-01)** | 1,191,859 | 1,270,025 |
| **Waiting time > 0 and < 1096 days** | 1,167,002 | 1,246,142 |
| **Non-missing and valid deprivation quintile and purchase code** | 1,151,006 | 1,231,854 |

**Table ST2. NHS England Funded Elective Primary Hip and Knee Replacement Admissions 01 April 1997 to 31 March 2019 by Period and Provider**

|  | **Hip Replacements** |  |  | **Knee Replacements** |  |  |
| --- | --- | --- | --- | --- | --- | --- |
|  | **NHS** | **Private Providers** | **TOTAL** | **NHS** | **Private Providers** | **TOTAL** |
| **Period 1 (Apr 1997 - Dec 2002)** | 203,072 (100%) | 0 (0%) | 203,072 | 177,487 (100%) | 0 (0%) | 177,487 |
| **Period 2 (Jan 2003 - Dec 2005)** | 137,817 (98.54%) | 2,049 (1.46%) | 139,866 | 152,013 (98.32%) | 2,594 (1.68%) | 154,607 |
| **Period 3 (Jan 2006 – Mar 2008)** | 115,096 (96.12%) | 4,648 (3.88%) | 119,744 | 131,860 (96.06%) | 5,414 (3.94%) | 137,274 |
| **Period 4 (Apr 2008 - Mar 2019)** | 557,508 (79.16%) | 146,812 (20.84%) | 704,320 | 615,478 (79.24%) | 161,296 (20.76%) | 776,774 |
| **TOTAL** | **1,013,493 (86.85%)** | **153,509 (13.15%)** | **1,167,002** | 1,076,838 (86.41%) | 169,304 (13.59%) | 1,246,142 |

**Table ST3. NHS England Funded Elective Primary Hip Replacement Admissions 01 April 1997 to 31 March 2019. Admissions and Admission Rates by Financial Year and Provider**

|  |  | **All Providers** |  | **NHS** |  | **Private Providers** |  |
| --- | --- | --- | --- | --- | --- | --- | --- |
| **Financial Year** | **Population (all ages)** | **Admissions** | **Admission Rate** | **Admissions** | **Admission Rate** | **Admissions** | **Admission Rate** |
| **1997/98** | 48,664,800 | 30,606 | 62.9 | 30,606 | 62.9 | 0 | 0 |
| **1998/99** | 48,820,600 | 33,966 | 69.6 | 33,966 | 69.6 | 0 | 0 |
| **1999/00** | 49,032,900 | 34,338 | 70.0 | 34,338 | 70.0 | 0 | 0 |
| **2000/01** | 49,233,300 | 36,162 | 73.5 | 36,162 | 73.5 | 0 | 0 |
| **2001/02** | 49,449,746 | 37,295 | 75.4 | 37,295 | 75.4 | 0 | 0 |
| **2002/03** | 49,679,267 | 41,803 | 84.1 | 41,773 | 84.1 | 30 | 0.1 |
| **2003/04** | 49,925,517 | 46,116 | 92.4 | 45,756 | 91.6 | 360 | 0.7 |
| **2004/05** | 50,194,600 | 46,467 | 92.6 | 45,247 | 90.1 | 1,220 | 2.4 |
| **2005/06** | 50,606,034 | 48,250 | 95.3 | 47,708 | 94.3 | 542 | 1.1 |
| **2006/07** | 50,965,186 | 51,405 | 100.9 | 49,779 | 97.7 | 1,626 | 3.2 |
| **2007/08** | 51,381,093 | 56,274 | 109.5 | 53,355 | 103.8 | 2,919 | 5.7 |
| **2008/09** | 51,815,853 | 58,581 | 113.1 | 52,616 | 101.5 | 5,965 | 11.5 |
| **2009/10** | 52,196,381 | 57,967 | 111.1 | 51,258 | 98.2 | 6,709 | 12.9 |
| **2010/11** | 52,642,452 | 60,651 | 115.2 | 50,727 | 96.4 | 9,924 | 18.9 |
| **2011/12** | 53,107,169 | 63,360 | 119.3 | 52,242 | 98.4 | 11,118 | 20.9 |
| **2012/13** | 53,493,729 | 61,893 | 115.7 | 50,782 | 94.9 | 11,111 | 20.8 |
| **2013/14** | 53,865,817 | 65,521 | 121.6 | 52,530 | 97.5 | 12,991 | 24.1 |
| **2014/15** | 54,316,618 | 65,594 | 120.8 | 51,705 | 95.2 | 13,889 | 25.6 |
| **2015/16** | 54,786,327 | 63,964 | 116.8 | 49,075 | 89.6 | 14,889 | 27.2 |
| **2016/17** | 55,268,067 | 68,007 | 123.0 | 49,999 | 90.5 | 18,008 | 32.6 |
| **2017/18** | 55,619,430 | 67,385 | 121.2 | 46,676 | 83.9 | 20,709 | 37.2 |
| **2018/19** | 55,977,178 | 71,397 | 127.5 | 49,898 | 89.1 | 21,499 | 38.4 |
| **TOTAL** |  | **1,167,002** |  | **1,013,493** |  | **153,509** |  |

**Table ST4. NHS England Funded Elective Primary Knee Replacement Admissions 01 April 1997 to 31 March 2019. Admissions and Admission Rates by Financial Year and Provider**

|  |  | **All Providers** |  | **NHS** |  | **Private Providers** |  |
| --- | --- | --- | --- | --- | --- | --- | --- |
| **Financial Year** | **Population (all ages)** | **Admissions** | **Admission Rate** | **Admissions** | **Admission Rate** | **Admissions** | **Admission Rate** |
| **1997/98** | 48,664,800 | 23,962 | 49.2 | 23,962 | 49.2 | 0 | 0 |
| **1998/99** | 48,820,600 | 27,992 | 57.3 | 27,992 | 57.3 | 0 | 0 |
| **1999/00** | 49,032,900 | 29,081 | 59.3 | 29,081 | 59.3 | 0 | 0 |
| **2000/01** | 49,233,300 | 32,133 | 65.3 | 32,133 | 65.3 | 0 | 0 |
| **2001/02** | 49,449,746 | 34,513 | 69.8 | 34,513 | 69.8 | 0 | 0 |
| **2002/03** | 49,679,267 | 41,442 | 83.4 | 41,396 | 83.3 | 46 | 0.1 |
| **2003/04** | 49,925,517 | 49,095 | 98.3 | 48,649 | 97.4 | 446 | 0.9 |
| **2004/05** | 50,194,600 | 52,213 | 104.0 | 50,647 | 100.9 | 1,566 | 3.1 |
| **2005/06** | 50,606,034 | 55,411 | 109.5 | 54,735 | 108.2 | 676 | 1.3 |
| **2006/07** | 50,965,186 | 58,278 | 114.3 | 56,353 | 110.6 | 1,925 | 3.8 |
| **2007/08** | 51,381,093 | 65,248 | 127.0 | 61,899 | 120.5 | 3,349 | 6.5 |
| **2008/09** | 51,815,853 | 68,097 | 131.4 | 61,201 | 118.1 | 6,896 | 13.3 |
| **2009/10** | 52,196,381 | 64,810 | 124.2 | 56,957 | 109.1 | 7,853 | 15.0 |
| **2010/11** | 52,642,452 | 66,583 | 126.5 | 55,652 | 105.7 | 10,931 | 20.8 |
| **2011/12** | 53,107,169 | 70,097 | 132.0 | 57,806 | 108.8 | 12,291 | 23.1 |
| **2012/13** | 53,493,729 | 67,593 | 126.4 | 55,837 | 104.4 | 11,756 | 22.0 |
| **2013/14** | 53,865,817 | 70,595 | 131.1 | 57,034 | 105.9 | 13,561 | 25.2 |
| **2014/15** | 54,316,618 | 72,188 | 132.9 | 57,001 | 104.9 | 15,187 | 28.0 |
| **2015/16** | 54,786,327 | 71,054 | 129.7 | 54,087 | 98.7 | 16,967 | 31.0 |
| **2016/17** | 55,268,067 | 76,569 | 138.5 | 56,107 | 101.5 | 20,462 | 37.0 |
| **2017/18** | 55,619,430 | 73,524 | 132.2 | 51,080 | 91.8 | 22,444 | 40.4 |
| **2018/19** | 55,977,178 | 75,664 | 135.2 | 52,716 | 94.2 | 22,948 | 41.0 |
| **TOTAL** |  | **1,246,142** |  | **1,076,838** |  | **169,304** |  |

**Table ST5 – NHS England Funded Elective Hip Replacement Admissions 01 April 1997 to 31 March 2019. Odds of Admission by Period and by Deprivation and Comorbidity**

|  | **Deprivation** |  |  | **Comorbidity** |  |  |
| --- | --- | --- | --- | --- | --- | --- |
|  | **IMD1 (most deprived)** | **IMD5 (least deprived)** | **Odds IMD1 vs. IMD5** | **One or more** | **None** | **Odds one or more vs. none** |
| **NHS** |  |  |  |  |  |  |
| **Period 1 (Apr 1997 - Dec 2002)** | 31,066 | 40,339 | 0.77 | 28,694 | 174,378 | 0.16 |
| **Period 2 (Jan 2003 - Dec 2005)** | 19,396 | 28,765 | 0.67 | 24,277 | 113,540 | 0.21 |
| **Period 3 (Jan 2006 – Mar 2008)** | 15,505 | 25,120 | 0.62 | 24,701 | 90,395 | 0.27 |
| **Period 4 (Apr 2008 - Mar 2019)** | 76,709 | 122,454 | 0.63 | 185,779 | 371,729 | 0.50 |
|  |  |  |  |  |  |  |
| **Private Providers** |  |  |  |  |  |  |
| **Period 1 (Apr 1997 - Dec 2002)** | no observations | no observations | no observations | no observations | no observations | no observations |
| **Period 2 (Jan 2003 - Dec 2005)** | 281 | 428 | 0.66 | 58 | 1,991 | 0.03 |
| **Period 3 (Jan 2006 – Mar 2008)** | 526 | 1,340 | 0.39 | 110 | 4,538 | 0.02 |
| **Period 4 (Apr 2008 - Mar 2019)** | 13,011 | 38,788 | 0.34 | 35,171 | 111,641 | 0.32 |

**Table ST6 – NHS England Funded Elective Knee Replacement Admissions 01 April 1997 to 31 March 2019. Odds of Admission by Period and by Deprivation and Comorbidity**

|  | **Deprivation** |  |  | **Comorbidity** |  |  |
| --- | --- | --- | --- | --- | --- | --- |
|  | **IMD1 (most deprived)** | **IMD5 (least deprived)** | **Odds IMD1 vs. IMD5** | **One or more** | **None** | **Odds one or more vs. none** |
| **NHS** |  |  |  |  |  |  |
| **Period 1 (Apr 1997 - Dec 2002)** | 31,599 | 31,913 | 0.99 | 31,104 | 146,383 | 0.21 |
| **Period 2 (Jan 2003 - Dec 2005)** | 24,522 | 29,623 | 0.83 | 32,184 | 119,829 | 0.27 |
| **Period 3 (Jan 2006 – Mar 2008)** | 20,467 | 26,591 | 0.77 | 33,746 | 98,114 | 0.34 |
| **Period 4 (Apr 2008 - Mar 2019)** | 102,349 | 120,650 | 0.85 | 231,670 | 383,808 | 0.60 |
|  |  |  |  |  |  |  |
| **Private Providers** |  |  |  |  |  |  |
| **Period 1 (Apr 1997 - Dec 2002)** | no observations | no observations | no observations | no observations | no observations | no observations |
| **Period 2 (Jan 2003 - Dec 2005)** | 364 | 505 | 0.72 | 120 | 2,474 | 0.05 |
| **Period 3 (Jan 2006 – Mar 2008)** | 776 | 1,354 | 0.57 | 176 | 5,238 | 0.03 |
| **Period 4 (Apr 2008 - Mar 2019)** | 18,037 | 38,719 | 0.47 | 45,547 | 115,749 | 0.39 |

**Table ST7 – NHS England Funded Elective Hip Replacement Admissions 01 April 1997 to 31 March 2019. Odds of Admission by Financial Year and by Deprivation and Comorbidity**

|  | **Deprivation** |  |  | **Comorbidity** |  |  |
| --- | --- | --- | --- | --- | --- | --- |
|  | **IMD1 (most deprived)** | **IMD5 (least deprived)** | **Odds IMD1 vs. IMD5** | **One or more** | **None** | **Odds one or more vs. none** |
| **NHS** |  |  |  |  |  |  |
| **1997/98** | 4,663 | 5,919 | 0.79 | 3,987 | 26,619 | 0.15 |
| **1998/99** | 5,278 | 6,611 | 0.80 | 4,633 | 29,333 | 0.16 |
| **1999/00** | 5,284 | 7,032 | 0.75 | 4,725 | 29,613 | 0.16 |
| **2000/01** | 5,541 | 7,256 | 0.76 | 5,303 | 30,859 | 0.17 |
| **2001/02** | 5,673 | 7,379 | 0.77 | 5,417 | 31,878 | 0.17 |
| **2002/03** | 6,268 | 8,374 | 0.75 | 6,297 | 35,476 | 0.18 |
| **2003/04** | 6,621 | 9,370 | 0.71 | 7,269 | 38,487 | 0.19 |
| **2004/05** | 6,294 | 9,480 | 0.66 | 8,258 | 36,989 | 0.22 |
| **2005/06** | 6,524 | 10,193 | 0.64 | 9,446 | 38,262 | 0.25 |
| **2006/07** | 6,620 | 10,966 | 0.60 | 10,374 | 39,405 | 0.26 |
| **2007/08** | 7,201 | 11,644 | 0.62 | 11,963 | 41,392 | 0.29 |
| **2008/09** | 7,116 | 11,656 | 0.61 | 12,782 | 39,834 | 0.32 |
| **2009/10** | 6,723 | 11,395 | 0.59 | 13,751 | 37,507 | 0.37 |
| **2010/11** | 7,056 | 11,053 | 0.64 | 15,063 | 35,664 | 0.42 |
| **2011/12** | 7,258 | 11,436 | 0.63 | 16,027 | 36,215 | 0.44 |
| **2012/13** | 6,995 | 11,491 | 0.61 | 16,311 | 34,471 | 0.47 |
| **2013/14** | 7,337 | 11,355 | 0.65 | 18,272 | 34,258 | 0.53 |
| **2014/15** | 7,300 | 11,291 | 0.65 | 18,119 | 33,586 | 0.54 |
| **2015/16** | 6,875 | 10,687 | 0.64 | 18,213 | 30,862 | 0.59 |
| **2016/17** | 6,866 | 11,078 | 0.62 | 19,082 | 30,917 | 0.62 |
| **2017/18** | 6,307 | 10,171 | 0.62 | 18,369 | 28,307 | 0.65 |
| **2018/19** | 6,876 | 10,841 | 0.63 | 19,790 | 30,108 | 0.66 |
| **Private Providers** |  |  |  |  |  |  |
| **1997/98** |  |  |  |  |  |  |
| **1998/99** |  |  |  |  |  |  |
| **1999/00** |  |  |  |  |  |  |
| **2000/01** |  |  |  |  |  |  |
| **2001/02** |  |  |  |  |  |  |
| **2002/03** |  |  |  |  |  |  |
| **2003/04** | 32 | 94 | 0.34 | 28 | 332 | 0.08 |
| **2004/05** | 156 | 246 | 0.63 | 23 | 1,197 | 0.02 |
| **2005/06** | 105 | 117 | 0.90 | 3 | 539 | 0.01 |
| **2006/07** | 210 | 470 | 0.45 | 3 | 1,623 | 0.00 |
| **2007/08** | 301 | 833 | 0.36 | 107 | 2,812 | 0.04 |
| **2008/09** | 600 | 1,425 | 0.42 | 648 | 5,317 | 0.12 |
| **2009/10** | 560 | 1,764 | 0.32 | 1,103 | 5,606 | 0.20 |
| **2010/11** | 945 | 2,555 | 0.37 | 1,930 | 7,994 | 0.24 |
| **2011/12** | 962 | 2,847 | 0.34 | 2,245 | 8,873 | 0.25 |
| **2012/13** | 945 | 2,949 | 0.32 | 2,352 | 8,759 | 0.27 |
| **2013/14** | 1,060 | 3,529 | 0.30 | 2,960 | 10,031 | 0.30 |
| **2014/15** | 1,236 | 3,680 | 0.34 | 3,273 | 10,616 | 0.31 |
| **2015/16** | 1,307 | 3,954 | 0.33 | 3,667 | 11,222 | 0.33 |
| **2016/17** | 1,510 | 4,793 | 0.32 | 4,622 | 13,386 | 0.35 |
| **2017/18** | 1,911 | 5,515 | 0.35 | 6,017 | 14,692 | 0.41 |
| **2018/19** | 1,975 | 5,777 | 0.34 | 6,354 | 15,145 | 0.42 |

**Table ST8 – NHS England Funded Elective Knee Replacement Admissions 01 April 1997 to 31 March 2019. Odds of Admission by Financial Year and by Deprivation and Comorbidity**

|  | **Deprivation** |  |  | **Comorbidity** |  |  |
| --- | --- | --- | --- | --- | --- | --- |
|  | **IMD1 (most deprived)** | **IMD5 (least deprived)** | **Odds IMD1 vs. IMD5** | **One or more** | **None** | **Odds one or more vs. none** |
| **NHS** |  |  |  |  |  |  |
| **1997/98** | 4,351 | 4,016 | 1.08 | 4,064 | 19,898 | 0.20 |
| **1998/99** | 5,265 | 4,902 | 1.07 | 4,771 | 23,221 | 0.21 |
| **1999/00** | 5,322 | 5,250 | 1.01 | 5,077 | 24,004 | 0.21 |
| **2000/01** | 5,605 | 5,766 | 0.97 | 5,706 | 26,427 | 0.22 |
| **2001/02** | 6,035 | 6,325 | 0.95 | 6,050 | 28,463 | 0.21 |
| **2002/03** | 6,892 | 7,871 | 0.88 | 7,551 | 33,845 | 0.22 |
| **2003/04** | 7,842 | 9,461 | 0.83 | 9,347 | 39,302 | 0.24 |
| **2004/05** | 8,293 | 9,845 | 0.84 | 11,036 | 39,611 | 0.28 |
| **2005/06** | 8,641 | 10,789 | 0.80 | 12,994 | 41,741 | 0.31 |
| **2006/07** | 8,768 | 11,369 | 0.77 | 14,203 | 42,150 | 0.34 |
| **2007/08** | 9,574 | 12,533 | 0.76 | 16,235 | 45,664 | 0.36 |
| **2008/09** | 9,981 | 12,177 | 0.82 | 17,446 | 43,755 | 0.40 |
| **2009/10** | 9,224 | 11,260 | 0.82 | 17,852 | 39,105 | 0.46 |
| **2010/11** | 9,449 | 10,777 | 0.88 | 19,034 | 36,618 | 0.52 |
| **2011/12** | 9,788 | 11,354 | 0.86 | 20,536 | 37,270 | 0.55 |
| **2012/13** | 9,464 | 10,862 | 0.87 | 20,547 | 35,290 | 0.58 |
| **2013/14** | 9,817 | 10,912 | 0.90 | 22,549 | 34,485 | 0.65 |
| **2014/15** | 9,826 | 10,928 | 0.90 | 22,828 | 34,173 | 0.67 |
| **2015/16** | 9,014 | 10,612 | 0.85 | 22,250 | 31,837 | 0.70 |
| **2016/17** | 9,059 | 11,299 | 0.80 | 23,680 | 32,427 | 0.73 |
| **2017/18** | 8,254 | 10,082 | 0.82 | 21,847 | 29,233 | 0.75 |
| **2018/19** | 8,473 | 10,387 | 0.82 | 23,101 | 29,615 | 0.78 |
| **Private Providers** |  |  |  |  |  |  |
| **1997/98** |  |  |  |  |  |  |
| **1998/99** |  |  |  |  |  |  |
| **1999/00** |  |  |  |  |  |  |
| **2000/01** |  |  |  |  |  |  |
| **2001/02** |  |  |  |  |  |  |
| **2002/03** |  |  |  |  |  |  |
| **2003/04** | 37 | 117 | 0.32 | 50 | 396 | 0.13 |
| **2004/05** | 227 | 302 | 0.75 | 58 | 1,508 | 0.04 |
| **2005/06** | 122 | 106 | 1.15 | 7 | 669 | 0.01 |
| **2006/07** | 295 | 523 | 0.56 | 1 | 1,924 | 0.00 |
| **2007/08** | 454 | 801 | 0.57 | 175 | 3,174 | 0.06 |
| **2008/09** | 841 | 1,472 | 0.57 | 955 | 5,941 | 0.16 |
| **2009/10** | 800 | 1,899 | 0.42 | 1,520 | 6,333 | 0.24 |
| **2010/11** | 1,196 | 2,644 | 0.45 | 2,634 | 8,297 | 0.32 |
| **2011/12** | 1,428 | 2,835 | 0.50 | 2,995 | 9,296 | 0.32 |
| **2012/13** | 1,278 | 2,805 | 0.46 | 2,969 | 8,787 | 0.34 |
| **2013/14** | 1,450 | 3,289 | 0.44 | 3,650 | 9,911 | 0.37 |
| **2014/15** | 1,640 | 3,642 | 0.45 | 4,255 | 10,932 | 0.39 |
| **2015/16** | 1,936 | 4,053 | 0.48 | 4,901 | 12,066 | 0.41 |
| **2016/17** | 2,391 | 4,821 | 0.50 | 6,309 | 14,153 | 0.45 |
| **2017/18** | 2,554 | 5,576 | 0.46 | 7,502 | 14,942 | 0.50 |
| **2018/19** | 2,523 | 5,683 | 0.44 | 7,857 | 15,091 | 0.52 |

**2.3. Waiting times**

**Table ST9. NHS England Funded Elective Primary Hip and Knee Replacement Mean Waiting Times in Days 01 April 1997 to 31 March 2019 by Period and Provider**

|  | **Hip Replacements** |  |  | **Knee Replacements** |  |  |
| --- | --- | --- | --- | --- | --- | --- |
|  | **NHS** | **Private Provider** | **All Admissions** | **NHS** | **Private Provider** | **All Admissions** |
| **Period 1 (Apr 1997 - Dec 2002)** | 238.1 | no observations | 238.1 | 283.6 | no observations | 283.6 |
| **Period 2 (Jan 2003 - Dec 2005)** | 199.8 | 119.2 | 198.6 | 221.6 | 122.6 | 220.0 |
| **Period 3 (Jan 2006 – Mar 2008)** | 135.1 | 67.1 | 132.5 | 146.1 | 69.2 | 143.1 |
| **Period 4 (Apr 2008 - Mar 2019)** | 96.9 | 61.4 | 89.5 | 102.6 | 69.1 | 95.7 |

**Table ST10. NHS England Funded Elective Primary Hip and Knee Replacement Admissions 01 April 1997 to 31 March 2019. Mean Waiting Times by Financial Year and Provider**

| **Financial Year** | **Hip Replacements** | | | **Knee Replacements** | | |
| --- | --- | --- | --- | --- | --- | --- |
|  | **All** | **NHS** | **Private Provider** | **All** | **NHS** | **Private Provider** |
| **1997/98** | 217.8 | 217.8 | Not available | 265.0 | 265.0 | Not available |
| **1998/99** | 232.7 | 232.7 | Not available | 283.7 | 283.7 | Not available |
| **1999/00** | 234.9 | 234.9 | Not available | 283.2 | 283.2 | Not available |
| **2000/01** | 246.8 | 246.8 | Not available | 292.3 | 292.3 | Not available |
| **2001/02** | 250.2 | 250.2 | Not available | 292.1 | 292.1 | Not available |
| **2002/03** | 243.9 | 243.8 | Not available* | 279.7 | 279.6 | Not available* |
| **2003/04** | 221.8 | 220.9 | 332.0 | 249.0 | 248.2 | 340.4 |
| **2004/05** | 187.4 | 190.5 | 74.5 | 206.1 | 210.1 | 74.8 |
| **2005/06** | 164.9 | 166.2 | 56.8 | 181.8 | 183.4 | 58.7 |
| **2006/07** | 147.2 | 150.1 | 58.2 | 159.3 | 162.7 | 59.2 |
| **2007/08** | 114.5 | 116.8 | 71.6 | 123.3 | 126.0 | 74.8 |
| **2008/09** | 84.4 | 85.8 | 72.3 | 90.0 | 90.6 | 85.2 |
| **2009/10** | 83.4 | 87.7 | 50.5 | 88.2 | 91.6 | 63.9 |
| **2010/11** | 83.4 | 90.4 | 47.6 | 88.6 | 95.2 | 55.3 |
| **2011/12** | 88.7 | 96.1 | 54.3 | 95.4 | 102.5 | 62 |
| **2012/13** | 83.4 | 90.4 | 51.4 | 90.3 | 96.7 | 59.7 |
| **2013/14** | 82.4 | 89.6 | 53.4 | 88.7 | 95 | 62.2 |
| **2014/15** | 84.2 | 90.7 | 60.3 | 89.5 | 95.7 | 66.2 |
| **2015/16** | 87.1 | 93.8 | 64.9 | 92.9 | 99.5 | 71.8 |
| **2016/17** | 95.3 | 106.5 | 64.3 | 101.1 | 112.6 | 69.6 |
| **2017/18** | 98.7 | 112.8 | 67.0 | 105.9 | 120 | 73.8 |
| **2018/19** | 108.9 | 124.8 | 71.9 | 117.9 | 134.1 | 80.5 |

* there were 30 and 11 hip and knee replacement admissions to private providers respectively in the last quarter of 2002/03

**2.4. Modelling waiting time**

**Table ST11 – NHS England Funded Elective Primary Hip and Knee Replacement Admissions 01 April 1997 to 31 March 2019. Results from Interrupted Time Series Models**

|  | | **Hip Replacement** | **Knee Replacement** |
| --- | --- | --- | --- |
| **Trend** | **01 April 1997 to 31 December 2002** | -1.16 | -1.83 |
|  | **01 January 2003 to 31 March 2019** | 0.29 | 0.32 |
| **Level change** | **01 January 2003** | -2.24 (-10.42, 5.95) | 16.63 (12.64, 20.62) |
| **Trend Change** | **01 January 2003** | 1.45 (0.98, 1.92) | 2.14 (1.89, 2.40) |

**Table ST12 – NHS England Funded Elective Hip Replacement Admissions Interaction Provider vs. Deprivation. Waiting Time Ratios with 95% Confidence Intervals and Relative Percentage Change from Loglogistic Accelerated Failure Time Survival Model**

|  |  | **Time Ratios** | **Relative Percentage Change** |
| --- | --- | --- | --- |
| **IMD1 vs. IMD5** | **NHS** | 1.0190 (1.0138, 1.0243) | 1.90% |
|  | **Private Providers** | 1.0492 (1.0333, 1.0655) | 4.92% |
| **Provider** | **NHS** | 1 | 0 |
|  | **Private Providers** | 0.5025 (0.4980, 0.5072) | -49.75% |
| **Admission Rate per Day** |  | 0.9888 (0.9887, 0.9889) | -1.12% |
| **Private Provider Share** |  | 1.0193 (1.0188, 1.0198) | 1.93% |
| **Period 1** | **Apr 1997 - Dec 2002** | 1 | 0 |
| **Period 2** | **Jan 2003 - Dec 2005** | 1.0770 (1.0690, 1.0849) | 7.70% |
| **Period 3** | **Jan 2006 – Mar 2008** | 0.8729 (0.8652, 0.8806) | -12.71% |
| **Period 4** | **Apr 2008 - Mar 2019** | 0.6447 (0.6374, 0.6522) | -35.53% |
| **Area Level Socioeconomic Deprivation** | **IMD2** | 1.0129 (1.0083, 1.0175) | 1.29% |
|  | **IMD3** | 1.0093 (1.0051, 1.0136) | 0.93% |
|  | **IMD4** | 1.0009 (0.9968, 1.0049) | 0.09% |
|  | **IMD5 (least deprived)** | 1 | 0 |
| **Comorbidity** | **No comorbidity** | 1 | 0 |
|  | **One or more comorbidity** | 0.9995 (0.9965, 1.0024) | -0.05% |
| **Gender** | **Male** | 1.0190 (1.0138, 1.0243) | 1.90% |
|  | **Female** | 1.0492 (1.0333, 1.0655) | 4.92% |

**Table ST13 – NHS England Funded Elective Knee Replacement Admissions Interaction Provider vs. Deprivation. Waiting Time Ratios with 95% Confidence Intervals and Relative Percentage Change from Loglogistic Accelerated Failure Time Survival Model**

|  |  | **Time Ratios** | **Relative Percentage Change** |
| --- | --- | --- | --- |
| **IMD1 vs. IMD5** | **NHS** | 1.0077 (1.0030, 1.0124) | 0.77% |
|  | **Private Providers** | 1.0299 (1.0158, 1.0442) | 2.99% |
| **Provider** | **NHS** | 1 | 0 |
|  | **Private Providers** | 0.4793 (0.4750, 0.4837) | -52.07% |
| **Admission Rate per Day** |  | 0.9899 (0.9898, 0.9900) | -1.01% |
| **Private Provider Share** |  | 1.0135 (1.0131, 1.0140) | 1.35% |
| **Period 1** | **Apr 1997 - Dec 2002** | 1 | 0 |
| **Period 2** | **Jan 2003 - Dec 2005** | 1.1688 (1.1599, 1.1777) | 16.88% |
| **Period 3** | **Jan 2006 – Mar 2008** | 0.9985 (0.9891, 1.0079) | -0.15% |
| **Period 4** | **Apr 2008 - Mar 2019** | 0.7123 (0.7042, 0.7205) | -28.77% |
| **Area Level Socioeconomic Deprivation** | **IMD2** | 1.0072 (1.0030, 1.0115) | 0.72% |
|  | **IMD3** | 1.0044 (1.0004, 1.0084) | 0.44% |
|  | **IMD4** | 1.0023 (0.9984, 1.0062) | 0.23% |
|  | **IMD5 (least deprived)** | 1 | 0 |
| **Comorbidity** | **No comorbidity** | 1 | 0 |
|  | **One or more comorbidity** | 1.0144 (1.0117, 1.0170) | 1.44% |

**2.5. Number of sites and private treatment share**

**Table ST14 – NHS England Funded Elective Hip and Knee Replacement Admissions 01 January 2003 to 31 March 2019. Number of Private Provider Sites by Financial Year Quarter**

| **Financial Year** | **Quarter** | **Hip Replacements** | **Knee Replacements** |
| --- | --- | --- | --- |
| **2002/03** | **4** | 13 | 13 |
| **2003/04** | **1** | 15 | 16 |
|  | **2** | 13 | 14 |
|  | **3** | 14 | 14 |
|  | **4** | 9 | 12 |
| **2004/05** | **1** | 10 | 10 |
|  | **2** | 17 | 12 |
|  | **3** | 26 | 25 |
|  | **4** | 28 | 29 |
| **2005/06** | **1** | 17 | 17 |
|  | **2** | 4 | 4 |
|  | **3** | 3 | 4 |
|  | **4** | 4 | 6 |
| **2006/07** | **1** | 7 | 7 |
|  | **2** | 10 | 9 |
|  | **3** | 7 | 7 |
|  | **4** | 10 | 9 |
| **2007/08** | **1** | 13 | 14 |
|  | **2** | 13 | 14 |
|  | **3** | 18 | 14 |
|  | **4** | 24 | 24 |
| **2008/09** | **1** | 61 | 61 |
|  | **2** | 47 | 48 |
|  | **3** | 45 | 44 |
|  | **4** | 71 | 71 |
| **2009/10** | **1** | 60 | 55 |
|  | **2** | 51 | 49 |
|  | **3** | 53 | 45 |
|  | **4** | 70 | 68 |
| **2010/11** | **1** | 92 | 86 |
|  | **2** | 90 | 92 |
|  | **3** | 94 | 90 |
|  | **4** | 97 | 102 |
| **2011/12** | **1** | 94 | 97 |
|  | **2** | 102 | 101 |
|  | **3** | 106 | 106 |
|  | **4** | 106 | 110 |
| **2012/13** | **1** | 99 | 101 |
|  | **2** | 106 | 104 |
|  | **3** | 109 | 107 |
|  | **4** | 107 | 105 |
| **2013/14** | **1** | 107 | 110 |
|  | **2** | 107 | 106 |
|  | **3** | 105 | 105 |
|  | **4** | 107 | 111 |
| **2014/15** | **1** | 104 | 108 |
|  | **2** | 105 | 109 |
|  | **3** | 108 | 110 |
|  | **4** | 109 | 109 |
| **2015/16** | **1** | 111 | 111 |
|  | **2** | 112 | 111 |
|  | **3** | 112 | 114 |
|  | **4** | 115 | 119 |
| **2016/17** | **1** | 115 | 117 |
|  | **2** | 123 | 129 |
|  | **3** | 136 | 141 |
|  | **4** | 150 | 150 |
| **2017/18** | **1** | 154 | 157 |
|  | **2** | 159 | 156 |
|  | **3** | 156 | 159 |
|  | **4** | 156 | 156 |
| **2018/19** | **1** | 155 | 158 |
|  | **2** | 153 | 155 |
|  | **3** | 157 | 159 |
|  | **4** | 155 | 158 |

**Table ST15 – NHS England Funded Elective Hip and Knee Replacement Admissions 01 April 1997 to 31 March 2019. Share of Admissions to Private Providers by Financial Year Quarter**

| **Financial Year** | **Quarter** | **Hip replacements** | | | **Knee replacements** | | |
| --- | --- | --- | --- | --- | --- | --- | --- |
|  |  | **NHS** | **Private Providers** | **Private Share** | **NHS** | **Private Providers** | **Private Share** |
| **2002/03** | **4** | 11,068 | 30 | 0.3% | 11,590 | 46 | 0.4% |
| **2003/04** | **1** | 10,468 | 85 | 0.8% | 11,005 | 108 | 1.0% |
|  | **2** | 11,428 | 108 | 0.9% | 11,906 | 123 | 1.0% |
|  | **3** | 11,275 | 107 | 0.9% | 11,849 | 140 | 1.2% |
|  | **4** | 12,585 | 60 | 0.5% | 13,889 | 75 | 0.5% |
| **2004/05** | **1** | 11,555 | 158 | 1.3% | 12,620 | 172 | 1.3% |
|  | **2** | 11,622 | 233 | 2.0% | 12,681 | 255 | 2.0% |
|  | **3** | 10,843 | 394 | 3.5% | 12,140 | 556 | 4.4% |
|  | **4** | 11,227 | 435 | 3.7% | 13,206 | 583 | 4.2% |
| **2005/06** | **1** | 11,911 | 221 | 1.8% | 13,569 | 287 | 2.1% |
|  | **2** | 11,804 | 86 | 0.7% | 13,435 | 113 | 0.8% |
|  | **3** | 12,031 | 132 | 1.1% | 14,123 | 136 | 1.0% |
|  | **4** | 11,962 | 103 | 0.9% | 13,608 | 140 | 1.0% |
| **2006/07** | **1** | 12,145 | 226 | 1.8% | 13,788 | 226 | 1.6% |
|  | **2** | 11,949 | 348 | 2.8% | 13,312 | 366 | 2.7% |
|  | **3** | 12,145 | 464 | 3.7% | 13,477 | 573 | 4.1% |
|  | **4** | 13,540 | 588 | 4.2% | 15,776 | 760 | 4.6% |
| **2007/08** | **1** | 12,693 | 628 | 4.7% | 14,741 | 743 | 4.8% |
|  | **2** | 13,136 | 713 | 5.1% | 15,008 | 815 | 5.2% |
|  | **3** | 13,397 | 772 | 5.4% | 15,706 | 804 | 4.9% |
|  | **4** | 14,129 | 806 | 5.4% | 16,444 | 987 | 5.7% |
| **2008/09** | **1** | 13,604 | 1,489 | 9.9% | 15,365 | 1,633 | 9.6% |
|  | **2** | 12,854 | 1,353 | 9.5% | 15,194 | 1,657 | 9.8% |
|  | **3** | 13,205 | 1,490 | 10.1% | 15,304 | 1,665 | 9.8% |
|  | **4** | 12,953 | 1,633 | 11.2% | 15,338 | 1,941 | 11.2% |
| **2009/10** | **1** | 12,593 | 1,546 | 10.9% | 14,000 | 1,920 | 12.1% |
|  | **2** | 12,790 | 1,673 | 11.6% | 14,187 | 1,930 | 12.0% |
|  | **3** | 12,932 | 1,631 | 11.2% | 14,191 | 1,952 | 12.1% |
|  | **4** | 12,943 | 1,859 | 12.6% | 14,579 | 2,051 | 12.3% |
| **2010/11** | **1** | 12,879 | 2,397 | 15.7% | 14,028 | 2,713 | 16.2% |
|  | **2** | 12,892 | 2,478 | 16.1% | 14,290 | 2,714 | 16.0% |
|  | **3** | 12,176 | 2,510 | 17.1% | 13,269 | 2,742 | 17.1% |
|  | **4** | 12,780 | 2,539 | 16.6% | 14,065 | 2,762 | 16.4% |
| **2011/12** | **1** | 12,369 | 2,396 | 16.2% | 13,686 | 2,763 | 16.8% |
|  | **2** | 13,329 | 2,770 | 17.2% | 14,631 | 3,066 | 17.3% |
|  | **3** | 12,768 | 2,862 | 18.3% | 14,363 | 3,151 | 18.0% |
|  | **4** | 13,776 | 3,090 | 18.3% | 15,126 | 3,311 | 18.0% |
| **2012/13** | **1** | 12,614 | 2,663 | 17.4% | 13,960 | 2,773 | 16.6% |
|  | **2** | 12,837 | 2,686 | 17.3% | 13,887 | 2,821 | 16.9% |
|  | **3** | 12,857 | 2,872 | 18.3% | 14,126 | 2,924 | 17.1% |
|  | **4** | 12,474 | 2,890 | 18.8% | 13,864 | 3,238 | 18.9% |
| **2013/14** | **1** | 13,040 | 3,024 | 18.8% | 13,896 | 2,978 | 17.6% |
|  | **2** | 13,032 | 3,118 | 19.3% | 14,027 | 3,199 | 18.6% |
|  | **3** | 13,096 | 3,302 | 20.1% | 14,251 | 3,485 | 19.6% |
|  | **4** | 13,362 | 3,547 | 21.0% | 14,860 | 3,899 | 20.8% |
| **2014/15** | **1** | 12,863 | 3,197 | 19.9% | 13,710 | 3,432 | 20.0% |
|  | **2** | 13,507 | 3,260 | 19.4% | 14,981 | 3,588 | 19.3% |
|  | **3** | 12,818 | 3,550 | 21.7% | 14,446 | 3,967 | 21.5% |
|  | **4** | 12,517 | 3,882 | 23.7% | 13,864 | 4,200 | 23.3% |
| **2015/16** | **1** | 12,526 | 3,559 | 22.1% | 13,721 | 3,824 | 21.8% |
|  | **2** | 12,378 | 3,568 | 22.4% | 13,782 | 4,050 | 22.7% |
|  | **3** | 12,465 | 3,766 | 23.2% | 13,639 | 4,375 | 24.3% |
|  | **4** | 11,706 | 3,996 | 25.4% | 12,945 | 4,718 | 26.7% |
| **2016/17** | **1** | 12,676 | 4,060 | 24.3% | 14,077 | 4,440 | 24.0% |
|  | **2** | 12,714 | 4,053 | 24.2% | 14,176 | 4,476 | 24.0% |
|  | **3** | 12,375 | 4,654 | 27.3% | 13,902 | 5,377 | 27.9% |
|  | **4** | 12,234 | 5,241 | 30.0% | 13,952 | 6,169 | 30.7% |
| **2017/18** | **1** | 12,536 | 5,255 | 29.5% | 13,822 | 5,512 | 28.5% |
|  | **2** | 12,269 | 5,005 | 29.0% | 13,450 | 5,471 | 28.9% |
|  | **3** | 12,270 | 5,196 | 29.7% | 13,460 | 5,597 | 29.4% |
|  | **4** | 9,601 | 5,253 | 35.4% | 10,348 | 5,864 | 36.2% |
| **2018/19** | **1** | 12,831 | 5,092 | 28.4% | 13,674 | 5,356 | 28.1% |
|  | **2** | 12,613 | 5,189 | 29.1% | 12,986 | 5,407 | 29.4% |
|  | **3** | 12,989 | 5,610 | 30.2% | 13,675 | 5,951 | 30.3% |
|  | **4** | 11,465 | 5,608 | 32.8% | 12,381 | 6,234 | 33.5% |
| **TOTAL** |  | **1,013,493** | **153,509** | **13.2%** | **1,076,838** | **169,304** | **13.6%** |

**References**

1. ISD National Services NHS Scotland. Summary of OPCS codes used by Scottish Arthroplasty Project. http://www.arthro.scot.nhs.uk/OPCS_codes_summary_150710.pdf (accessed 24 Jan 2025).

2. Cooper ZN, McGuire A, Jones S, Le Grand J. Equity, waiting times, and NHS reforms: retrospective study. *Bmj* 2009; **339**: b3264.

3. Laudicella M, Siciliani L, Cookson R. Waiting times and socioeconomic status: evidence from England. *Soc Sci Med* 2012; **74**(9): 1331-41.

4. NHS Digital. Hospital Episode Statistics Data Dictionary. HES+TOS+V2.01. Admitted Patient Care (APC). 2024. https://digital.nhs.uk/data-and-information/data-tools-and-services/data-services/hospital-episode-statistics/hospital-episode-statistics-data-dictionary (accessed 24 Jan 2025).

5. Department for Levelling Up, Communities & Local Government. English indices of deprivation. 13 December 2012. https://www.gov.uk/government/collections/english-indices-of-deprivation (accessed 24 Jan 2025).

6. Armitage JN, van der Meulen JH, Royal College of Surgeons Co-morbidity Consensus G. Identifying co-morbidity in surgical patients using administrative data with the Royal College of Surgeons Charlson Score. *The British journal of surgery* 2010; **97**(5): 772-81.

7. Wagner AK, Soumerai SB, Zhang F, Ross-Degnan D. Segmented regression analysis of interrupted time series studies in medication use research. *J Clin Pharm Ther* 2002; **27**(4): 299-309.

8. Huitema BE. The Analysis of Covariance and Alternatives: Statistical Methods for Experiments, Quasi‐Experiments, and Single‐Case Studies, Second Edition: John Wiley & Sons, Inc.; 2011.

9. SAS. Chapter 8. The AUTOREG Procedure.

10. Collett D. Modelling survival data in Medical Research. Third Edition. 2015.
